# Taohong Siwu-Containing Serum Enhances Angiogenesis in Rat Aortic Endothelial Cells by Regulating the VHL/HIF-1*α*/VEGF Signaling Pathway

**DOI:** 10.1155/2021/6610116

**Published:** 2021-11-22

**Authors:** Zhi Tang, Wangyang Li, Hongzan Xie, Shengping Jiang, Yunqing Pu, Hui Xiong

**Affiliations:** ^**1**^ Department of Orthopedics, Xiangtan Chinese Medicine Hospital, Xiangtan 411100, China; ^2^Department of Clinical Medicine, Hunan University of Traditional Chinese Medicine, Changsha 410208, China

## Abstract

**Background:**

The incidence of bone fracture and bone-related diseases is increasing every year. Angiogenesis plays a vital role in fracture healing and bone repair. This study assessed the benefits of Taohong Siwu (TSW) decoction on angiogenesis in isolated rat aortic endothelial cells (RAEC) treated with TSW-containing serum.

**Methods:**

The components of TSW decoction were analyzed by liquid chromatography-mass spectrometry (LC-MS). TSW-containing serum was prepared by gavage of TSW decoction to Sprague-Dawley (SD) rats. The effects of TSW-containing serum on the viability, migration, wound healing, and angiogenesis of RAEC were detected by the MTT, transwell, wound healing, and Matrigel lumen formation assays, respectively. In addition, the effects of an HIF-1*α* inhibitor on TSW-containing serum-induced RAEC were also assessed. The effects of TSW-containing serum on the expression of the HIF-1*α* signaling pathway were evaluated by qRT-PCR and western blot analysis.

**Results:**

LC-MS revealed that TSW decoction primarily contained isomaltulose, choline, D-gluconic acid, L-pipecolic acid, hypotaurine, albiflorin, and tryptophan. TSW-containing serum significantly increased the viability, migration, wound healing, and angiogenesis of RAEC in a dose-dependent manner. Furthermore, our results demonstrated that HIF-1*α* and VEGF expressions were increased in the cells of TSW-containing serum groups, whereas VHL expression was decreased. The effects of TSW-containing serum were reversed by treatment with an HIF-1*α* inhibitor.

**Conclusion:**

These results suggested that TSW decoction enhanced angiogenesis by regulating the VHL/HIF-1*α*/VEGF signaling pathway.

## 1. Background

The incidence of bone fracture and bone-related diseases is increasing every year. Fracture healing and bone repair are complicated pathological processes [1]. Patients with bone-related diseases suffer from delayed healing following fracture treatment for some reasons, such as without proper vascularization and blood supply [1, 2]. Several factors in the local microenvironment, such as oxygen saturation, PH, cytokines, and growth factors, may significantly affect fracture healing via directly affecting blood vessel formation, proliferation and differentiation of bone cells, and the deposition of minerals [3]. Few studies on angiogenesis related to fracture healing and the development of related drugs are worthy of in-depth research.

Rat aortic endothelial cells (RAEC) were isolated from Sprague-Dawley (SD) rats, and then RAEC were identified and cultured. RAEC are commonly used to study cardiovascular diseases [4]. Wound healing and tissue regeneration are closely related to forming a sufficient blood vessel network [5]. The endothelial cell tubule formation could act as a marker of angiogenesis, which is also a prerequisite and basis for bone healing [6]. Proper angiogenesis is indispensable for fracture healing, so RAEC are also used to study fracture healing, bone tissue engineering, and so on [2]. In this study, RAEC were used to study the development of auxiliary drugs related to fracture healing.

Fractures are usually accompanied by the rupture and embolization of blood vessels, which causes local and systemic hemorheological changes, increasing blood viscosity, slowing local microcirculation blood flow, and decreasing tissue oxygen saturation [1]. Hypoxia might initiate the compensatory response and the transcription of hypoxia response genes (HRG) in the cell, such as vascular endothelial growth factor (VEGF), erythropoietin (EPO), tyrosine hydroxylase, and several enzymes involved in glycolysis [7, 8]. Studies have shown that such a response is dependent upon hypoxia-inducible factor-1*α* (HIF-1*α*) [9, 10]. Under hypoxic conditions, HIF-1*α* is upregulated as a central regulator of angiogenesis [11, 12]. It directly participates in the entire process of angiogenesis by altering the expression of related growth factors [9, 12–14]. In addition, Hippel–Lindau (VHL) has been shown to inhibit the accumulation of HIF-1*α* [15, 16].

It is well known that many traditional Chinese medicines (TCM) promote blood circulation and remove blood stasis. They are beneficial to the local and systemic blood circulation during the early stages of fracture healing, which may improve local blood oxygen status and create a good internal environment for fracture healing [17–20]. Taohong Siwu (TSW) decoction is one of the most popular TCM for promoting blood circulation, removing blood stasis, and treating orthopedic diseases [21, 22]. Previous studies have shown that TSW-containing serum enhances VEGF expression in human endothelial cells through the PI3K/Akt-eNOS axis [23]. TSW decoction promotes angiogenesis during early pregnancy in rats by increasing the expression of Ang-1/2 and Tie-2 [24]. However, the mechanism of TSW decoction on the regeneration of blood vessels and fracture healing remains unclear. In this study, we analyzed the components of TSW decoction and investigated the possible mechanism for its effects on angiogenesis.

## 2. Material and Methods

### 2.1. Animals and Breeding Environment

Twenty male SD rats weighing 200–260 g were purchased from the Hunan Slack Jingda Experimental Animal Co. Ltd. (license # SCXK (Hunan) 2013–0004). The rats were raised at the Experimental Animal Center of the Hunan University of Traditional Chinese Medicine. They were housed in a standard animal room at a temperature of 25°C ± 2°C and 50% ± 5% humidity with 12 h of light and dark cycle. The cages were cleaned once a week, and the litter was replaced as needed. Animals were provided free access to water and food provided by the Experimental Animal Center of the Hunan University of Traditional Chinese Medicine. Rats were adaptively fed for 1 week. All rats were randomly and equally divided into two groups, including a control group and a TSW decoction group. TSW decoction was administered to the stomach for 1 week. All the animal experiments and protocols were approved by the local ethics committee.

### 2.2. Preparation of TSW-Containing Serum

TSW decoction was prepared using *Semen Persicae*, *Flos Carthami*, *Angelica sinensis*, *Radix Paeoniae Alba*, *Rhizoma Chuanxiong*, and *Radix Rehmanniae Praeparata* at fixed proportions as previously described [25]. TSW decoction was extracted as described and concentrated to 1.13 g/mL [25]. Ten male SD rats were administered TSW decoction according to the following formula: rat dose (g/kg) = 6.25 × [adult dose (g)/adult body mass (60 kg)] × 3. In the TSW decoction group, rats were administered a dose of approximately 25 g/kg TSW-containing serum twice a day for 1 week, intragastrically. The remaining rats in the blank group were given the same volume of normal saline for 1 week. After 2 h of gavage, they were treated with 40 mg/kg pentobarbital. The blood was collected from the abdominal aorta, and 5 mL blood was collected and left at 4°C overnight. Following centrifugation, the serum was collected, sterilised by filtration using a 0.22 *μ*M filter, inactivated at 56°C, and stored at −80°C.

### 2.3. Liquid Chromatography-Mass Spectrometry (LC-MS)

TSW decoction (100 *μ*L) was dissolved in 900 *μ*L deionized water. The mixed solution was then ultrasonicated in an ice bath for 15 minutes. Finally, the mixed solution was centrifuged at 12,000 rpm for 10 min. The supernatant was filtered through a 0.45 *μ*m membrane. The collected samples were analyzed by LC-MS (UHPLC: Nexera UHPLC LC-30A, Shimadzu; MASS: TripleTOF5600+, AB SCIEX™), using a Shimadzu InertSustain C18 column (100 × 2.1 mm, 2 *μ*m). Mobile phases A and B for the LC were acetonitrile and 0.1% CH_3_COOH-H_2_O, respectively. The column temperature was 35°C, and the flow rate was 0.3 mL/min. The LC-MS conditions are shown in Table 1. Electrospray ionization (ESI) positive ion and negative ion modes were used for detection. The ESI source conditions included ion source gas 1 (Gas 1, 50 psi), ion source gas 2 (Gas 2, 50 psi), and curtain gas (CUR, 25 psi). The source temperature was 500°C (positive ion) and 450°C (negative ion). Ion Sapary Voltage Floating (ISVF) was 5,500 V (positive ion) and 4,400 V (negative ion). The TOF MS scan range was 100–1200 Da. The product ion scan range was 50–1,000 Da. The TOF MS scan accumulation time was 0.2 s, and the product ion scan accumulation time was 0.01 s. Information-dependent acquisition was used for the secondary MS using a high sensitivity mode. The declustering potential was ±60 V, and the collision energy was 35 ± 15 eV. The data were analyzed using Analysis Base File Converter and MS-DIAL 4.10 (Nature Methods, 12, 523–526, 2015) software. The extracted peak information was used to compare with the database and consisted of MassBank, ReSpect, and GNPS (14,951 records in total). In general, a total score greater than 80 was significant. The results are shown in Figure S1 and Table S1.

### 2.4. Isolation, Identification, and Culture of RAEC

The preparation of RAEC was based on isolation, identification, and culture previously described [2]. Briefly, dissection scissors were used to open the abdomen from the midline, and the abdominal aorta was exposed after blood perfusion. The aorta was stripped and digested to collect RAEC. The cells were collected, separated, and placed in a Petri dish, and the morphology of the cells was observed with an inverted fluorescence microscope.

### 2.5. Cell Treatment

RAEC were divided into five groups: a control group, a 2.5% TSW group, a 5% TSW group, a 10% TSW group, and a CAY10585 (an HIF-1*α* inhibitor) group. The control group cells were treated with 10% blank serum (0% TSW) for 24 h. The cells of the 2.5%, 5%, and 10% TSW groups were treated with 2.5% TSW-containing serum, 5% TSW-containing serum, and 10% TSW-containing serum, respectively, for 24 h. We calculated the mixture of different volumes of TSW-containing serum and blank serum to ensure that the concentration of blank serum used in all groups was 10%. The cells of the CAY10585 group were treated with 10% TSW-containing serum in combination with 20 *μ*M CAY10585 (cat# ab144422, Abcam, USA) for 24 h. To further verify the effects of TSW-containing serum, the cells were divided into six groups consisting of the control group, the 2.5% TSW group, the 5% TSW group, the 10% TSW group, the CAY10585 group, and a KC7F2 group (another HIF inhibitor-1, cat# S7946, Selleck, USA). The cells of the KC7F2 group were treated the same as that of the CAY10585 group.

### 2.6. Transwell Assay

A transwell chamber migration assay was used to examine the effects of TSW-containing serum and an HIF-1*α* inhibitor on cell migration. The cells were seeded into 24-well plates at 5 × 10^4^ cells/well. The cells were treated as described above. After treatment for 48 h, the cells at the bottom of the chamber were fixed with paraformaldehyde and then stained with 0.1% crystal violet. The migrated cells were observed and quantified with an inverted microscope. At least 5 different fields with more than 200 cells were counted, and the images were acquired.

### 2.7. MTT Assay

The MTT assay was used to detect the effects of TSW-containing serum on cell viability. Briefly, the cells were plated into 96-well plates at 3,000 cells/well and cultured for 24 h. Then, 100 *μ*L MTT solution (Cat#: M2128, Sigma, USA) was added and incubated for another 4 h. The solution was aspirated, and 100 *μ*L DMSO was added to each well. The absorbance was measured using a microplate reader at a wavelength of 490 nm.

### 2.8. Wound-Healing Assay

The cells were plated into 24-well plates and then treated with TSW-containing serum in the presence or absence of an HIF-1*α* inhibitor as described previously. Plates containing different groups of cells were scratched with a 20 *μ*L pipette to create a wound. The cells were washed with PBS buffer to remove the debris. Images were acquired at 0, 24, and 48 h after treatment to assess wound healing at 3 random locations. The wound area for each group was quantified.

### 2.9. Tube Formation Assay

Ninety-six-well plates were coated with 50 *μ*L Matrigel gel per well. The cells were then seeded into the 96-well plates at a density of 1.5 × 10^4^ cells/well. The cells were then treated with TSW-containing serum in the presence or absence of an HIF-1*α* inhibitor as described previously. After incubation for 6 h, tube formation was checked, and images were acquired with an inverted microscope. Images from at least 5 different fields were acquired using Image-Pro Plus 7.1 software to calculate the length of the lumen.

### 2.10. Quantitative Real-Time PCR (qRT-PCR)

Total RNA was extracted from treated cells and then reverse-transcribed into cDNA using reverse transcriptase (Cat#: K1622, Thermo Fisher, USA). The relative expression levels of HIF-1*α*, VEGF, and VHL were measured by qRT-PCR using a T100™ thermal cycler. GAPDH was used as endogenous control, and the relative expression of the target genes was calculated using the 2^−ΔΔCt^ method. The primers used for qRT-PCR are listed in Table 2.

### 2.11. Western Blot Analysis

Total protein was prepared from cells using lysis buffer (P0013, Beyotime Bio, China). Then, 30 *μ*g of protein from each group was separated by sodium dodecyl sulfate-polyacrylamide (SDS-PAGE) gels. Next, they were transferred onto polyvinylidene difluoride membranes (Millipore, USA). After blocking with 5% fat-free milk in PBS buffer, the membranes were incubated with anti-HIF-1*α* (Abcam, USA), VEGF (Abcam, USA), VHL (Abcam, USA), and *β*-actin (Proteintech, USA) antibodies overnight at 4°C. The membranes were then washed four times with PBST buffer for 5 min each time. The membranes were incubated with secondary antibodies for 1 h at room temperature. After washing with PBST buffer, protein bands were detected using enhanced chemiluminescence (Advans Group). The quantification of protein expression was done using ImageJ software (National Institutes of Health).

### 2.12. Statistical Analysis

All data were presented as the mean ± standard deviation from at least three independent experiments. The results were analyzed using GraphPad Prism 8 software (GraphPad Prism Software Inc., San Diego, USA). The statistical difference among groups was examined using a one-way ANOVA test, followed by Tukey's post hoc test. *P* < 0.05 was considered statistically significant.

## 3. Results

### 3.1. TSW Decoction Promoted Migration, whereas an HIF-1*α* Inhibitor Reversed the Effects

TSW decoction was prepared from *Semen Persicae*, *Flos Carthami*, *Angelica Sinensis*, *Radix Paeoniae Alba*, *Rhizoma Chuanxiong*, and *Radix Rehmanniae Praeparata* in fixed proportions as previously described [25]. To identify the specific components, we performed LC-MS experiments. The results indicated that TSW decoction primarily contained isomaltulose, choline, D-gluconic acid, L-pipecolic acid, hypotaurine, albiflorin, and tryptophan (Figure S1 and Table S1). The LS-MS results showed that TWS decoction contained ferulic acid and amygdalin, and their relative peak areas were 1,562,890 and 33,370, respectively. Previous studies indicated that the main active ingredients of TSW decoction were ferulic acid, paeoniflorin, amygdalin, hydroxysafflor yellow A, catalpol, and gallic acid [26, 27]. Therefore, we speculated that the main active components of TSW decoction were ferulic acid and amygdalin. Next, the rats were gavaged by TSW decoction and the acquired TSW-containing serum was used to treat RAEC.

**Figure 1:**
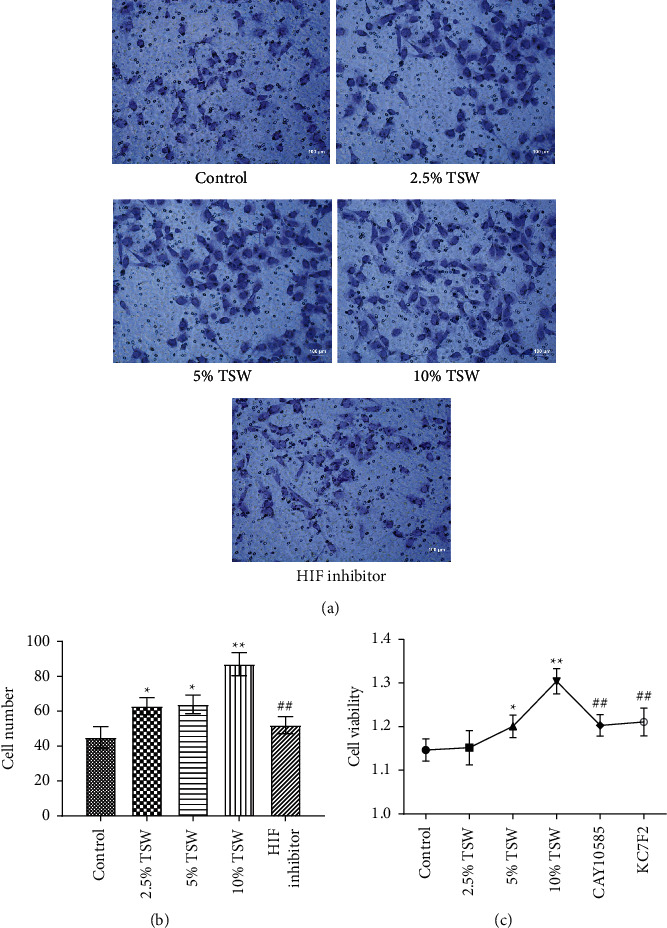
TSW decoction promoted migration, whereas an HIF-1*α* inhibitor reversed the effects: (a) the migrated cells were detected using a transwell assay; (b) the migrated cells were counted and quantified; and (c) the cell viability was measured by MTT assay. ^*∗∗*^*P* < 0.01 and ^*∗*^*P* < 0.05 versus the control group; ^##^*P* < 0.01 and ^#^*P* < 0.05 versus the 10% TSW group.

RAEC were isolated from rats. To investigate the effects of TSW decoction on cell migration, the transwell assay was performed. The results indicated that TSW-containing serum enhanced the migration of cells in a dose-dependent manner (Figures 1(a) and 1(b)). After the fracture, HIF-1*α* played an essential role in regulating hypoxia [28]. Therefore, we evaluated the effects of an HIF-1*α* inhibitor on TSW-containing serum-promoted migration. The results demonstrated that 10% TSW-containing serum-induced migration was blocked by HIF inhibitor treatment of the cells (Figures 1(a) and 1(b)). The cells were treated with different concentrations of TSW-containing serum, and the MTT assay measured cell viability. The results indicated that 5% and 10% TSW-containing serum significantly increased the viability of the cells compared with the control group, suggesting a therapeutic effect in cells after a fracture. In contrast, CAY10585 and KC7F2 decreased the viability of the cells compared with the 10% TSW group (Figure 1(c)).

### 3.2. TSW Decoction Expedited Migration, whereas HIF-1*α* Inhibition Reversed the Effects

**Figure 2:**
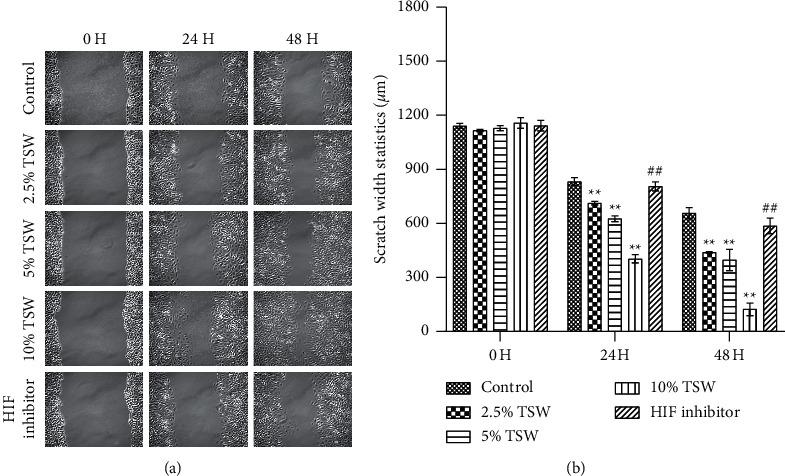
TSW decoction expedited migration, whereas HIF-1*α* inhibition reversed the effects: (a) migrated cells were detected using a wound-healing assay and (b) the wound area in each group was calculated.  ^*∗*^ ^*∗*^*P* < 0.01 versus the control group and ^##^*P* < 0.01 versus the 10% TSW group.

A wound-healing assay was carried out to examine the effects of TSW-containing serum on cell spreading. The results showed that the TSW-containing serum enhanced wound healing in a dose-dependent manner after the cells were treated for 24 and 48 h. HIF-1*α* inhibitor treatment reversed the effects of TSW-containing serum (Figure 2). These results suggested that TSW-containing serum regulated cell migration through HIF-1*α* signaling.

### 3.3. TSW Decoction Accelerated Angiogenesis, whereas HIF-1*α* Inhibition Decreased Angiogenesis

**Figure 3:**
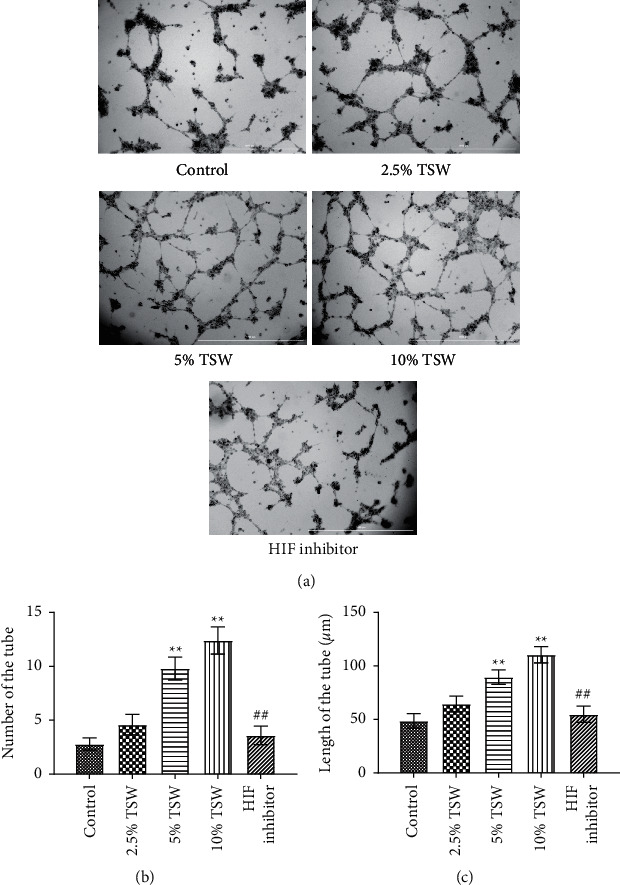
TSW decoction accelerated angiogenesis, whereas HIF-1*α* inhibition decreased angiogenesis: (a) angiogenesis was analyzed using an angiogenesis experiment, (b) the number of tubes in each group was quantified and analyzed, and (c) the length of the tube in each group was quantified.  ^*∗*^ ^*∗*^*P* < 0.01 versus the control group and ^##^*P* < 0.01 versus the 10% TSW group.

A tube formation assay was done to examine the effects of TSW-containing serum on RAEC angiogenesis. The results showed that TSW-containing serum enhanced tube formation in a dose-dependent manner. HIF-1*α* inhibitor treatment attenuated 10% TSW-containing serum-induced tube formation (Figure 3(a)). In addition, we measured the number of tubes and their length. The results showed that TSW-containing serum enhanced tube formation compared with the control group, whereas HIF-1*α* inhibition attenuated the effects on tube formation (Figures 3(b) and 3(c)). These results suggested that TSW-containing serum regulated angiogenesis by increasing tube formation in cells through HIF-1*α* signaling.

### 3.4. TSW-Containing Serum Regulated the HIF-1*α*/VEGF/VHL Signaling Pathway, whereas HIF-1*α* Inhibition Reversed the Effects

**Figure 4:**
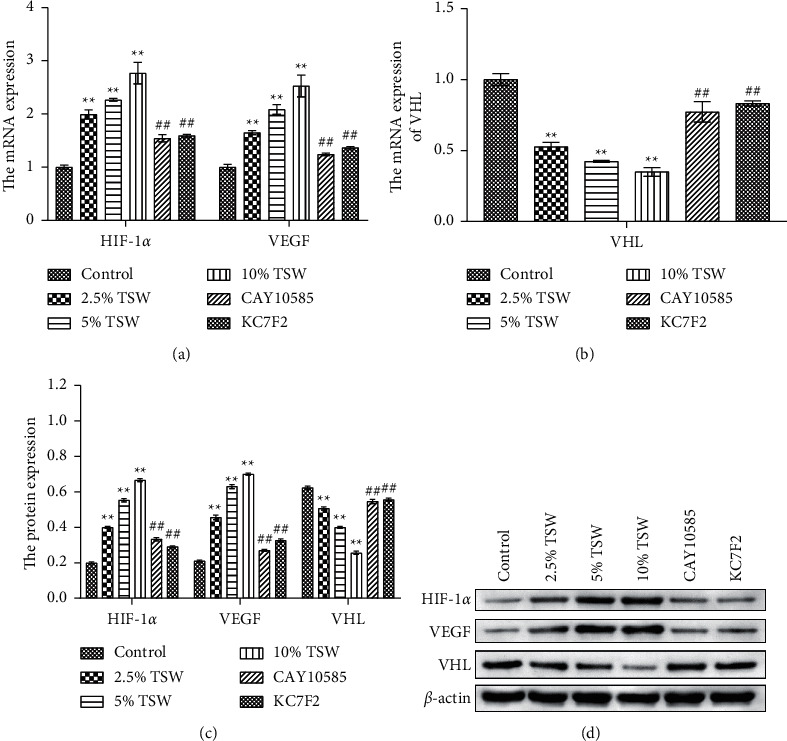
TSW decoction upregulated the HIF-1*α*/VEGF/VHL signaling pathway, whereas HIF-1*α* inhibitors reversed the effects: (a and b) the expression of HIF-1*α*, VEGF, and VHL mRNA was determined by qRT-PCR; (c) the expression of HIF-1*α*, VEGF, and VHL protein was quantified; and (d) the expression of HIF-1*α*, VEGF, and VHL protein was determined by western blot analysis.  ^*∗*^ ^*∗*^*P* < 0.01 versus the control group and ^##^*P* < 0.01versus the 10% TSW group.

To understand the role of HIF-1*α* signaling in the angiogenesis of RAEC, we measured the expression of HIF-1*α*, VEGF, and VHL mRNA. qRT-PCR results showed that the expression of HIF-1*α* and VEGF were increased, whereas VHL expression was decreased, and these effects were reversed with HIF-1*α* inhibitors (Figures 4(a) and 4(b)). Next, we measured the levels of HIF-1*α*, VEGF, and VHL protein by western blot analysis. The results showed that the levels of HIF-1*α* and VEGF protein were elevated, whereas VHL expression was suppressed. HIF-1*α* inhibitors attenuated these effects (Figures 4(c) and 4(d)). Altogether, the results demonstrated that TSW-containing serum regulated angiogenesis by activating the HIF-1*α*/VEGF/VHL signaling pathway.

## 4. Discussion

Previous studies have shown that TCM promotes blood circulation, removes blood stasis, accelerates bone fracture healing and angiogenesis in injured areas, and improves blood circulation at the injured site [17–20]. Bone healing usually involves four steps: (1) hematoma formation; (2) soft callus formation, including the proliferation of vascular endothelial cells; (3) hard callus formation, and (4) three-dimensional structure reconstruction of the lumen [29, 30]. In the early stage of bone fracture, local bleeding and hematoma occur [31]. Along with the absorption and mechanization of the hematoma, an exudate is produced. This exudate promotes the secretion of vascular proliferation regulators, thereby accelerating the sprouting of micro blood vessels, which differentiate into blood vessels [30, 31]. Angioplasty is the formation of tubules consisting of vascular endothelial cells. After the tubules are formed, they are interwoven into a network with one another and penetrate the membrane to promote the growth of fractured ends and induce bone structure remodeling [6]. Therefore, detection of vascular endothelial cell tubule formation as a marker of angiogenesis is also a prerequisite and basis for the healing process [6]. Our results showed that TSW-containing serum increased RAEC viability, indicating the efficacy of TSW decoction on RAEC growth. The results indicated that TSW-containing serum enhanced the angiogenesis of RAEC by increasing the migration, spreading, and tube formation. Angiogenesis is a double-edged sword. In cancer, angiogenesis often promotes the growth of cancer cells [32, 33]. However, in wound healing, angiogenesis is often beneficial [34–36]. For example, blue-green microalga *Spirulina platensis* and collagen could elevate the expression of angiogenesis-related factors bFGF and VEGF to promote wound healing [34, 35]. Adipose-derived stem cells increased diabetic wound healing by stimulating the release of angiogenic factors [36]. In our study, angiogenesis was increased after TSW-containing serum treatment. Angiogenesis might further have beneficial effects on fracture healing. We speculated that TSW might improve blood circulation at the injured site and further induce bone structure remodeling.

After the fracture, periosteal and vascular damage occurs at the fracture site, and the local blood supply is reduced or even interrupted, resulting in hypoxia. Bone tissue in a hypoxic environment can activate HIF-1*α*, thereby activating the HIF/VEGF signaling pathway, which participates in the vascular reconstruction of fracture ends and accelerates the fracture healing process [37, 38]. Next, we explored the underlying mechanism involved in TSW-containing serum-induced endothelial cell angiogenesis. Interestingly, our results showed that an HIF-1*α* inhibitor dramatically attenuated TSW-containing serum-induced angiogenesis by inhibiting the migration, spreading, and tube formation of RAEC, indicating the involvement of HIF-1*α* signaling. Previous studies showed that HIF-1*α* and VEGF expression are both upregulated during the healing process [14, 39]. In addition, the expression of HIF-1*α* mRNA is regulated by its upstream modulator VHL, which can induce the proteasome-dependent degradation of HIF-1*α* [39]. Recent studies have shown that the enhancement of angiogenesis and bone consolidation of some drugs during the process of osteogenesis may be related to the HIF-1*α*/VEGF/VHL signaling pathway [40, 41]. Our research showed that the TSW-containing serum enhanced angiogenesis by activating the HIF-1*α*/VEGF/VHL signaling pathway. Moreover, the inhibition of HIF-1*α* expression reversed the effects of TSW decoction and inhibited the expression of HIF-1*α*/VEGF.

Recent research showed that ferulic acid and amygdalin could promote bone fracture healing and stimulate blood circulation [42, 43]. Amygdalin effectively alleviates the inflammatory response in carrageenan-induced arthritis of rats [44]. The LS-MS results showed that TWS decoction contained ferulic acid and amygdalin, which might be the main active components. However, we cannot be sure of all the active ingredients at work in the TWS decoction. This was one of our limitations. We hope to carry out further experimental studies in the future to elaborate on the key substances in the TWS decoction. TSW-containing serum was obtained directly by gavage animals with TSW decoction. Because the gastrointestinal system of animals can selectively absorb and metabolize the components contained in TCM, the active components are probably similar, but their percentage may be different. The essence of the active ingredients was still from TSW decoction. We speculated that some of the main active ingredients of TSW decoction might be absorbed but could be detected in serum. For example, Liu et al. reported that songorine, benzoylhypaconitine, benzoylmesaconitine, and six other active ingredients were detected in the Yougui pill (YGP). Six of these components were also detected in the animal plasma of oral YGP in experimental autoimmune encephalomyelitis model rats. In addition, in vitro studies showed that YGP-containing serum and YGP extract had the same effects on the CREB/GAP-43 pathway [45]. Therefore, we speculated that TSW-containing serum might contain some effective components of TSW decoction. Because the serum compositions were too complex and the interference effects were too strong, the main components were detected in the decoction by LC-MS in many studies related to TCM. For example, the *Actinidia chinensis* Planch. and yam components were analyzed in gastric cancer and cartilage differentiation studies instead of the related drug-containing serum [46, 47]. Due to fund limitations, we did not research in depth on TSW-containing serum and TSW decoction components, which was a limitation of our research. However, we used blank serum as a control to eliminate the interference of serum. Essentially, the effects on the cells still come from the active ingredient of TSW decoction. We hope to carry out further experimental studies in the future to elaborate on the key substances and their in-depth mechanism of action in TSW decoction.

Based on the below reasons, TSW-containing serum was used for in vitro experiments. In vitro experiments are highly controlled and can reveal deeper mechanisms of action at the cellular, molecular, and gene levels. As is known to all, TCM is composed of complex and diverse chemical components. Their extracts contain both active components and a large number of ineffective components, some of which can affect and interfere with the in vitro experiments without specificity. The gastrointestinal system of the body can selectively absorb and metabolize the components contained in TCM so as to exert real pharmacological effects. Therefore, TCM decoction and pill cannot be directly added to in vitro cell culture. Many studies have shown that drug-containing rat serum is often used in vitro experiments [46–48]. In addition, the drug-containing rat serum has a good effect in simulated in vitro experiments. For example, the inhibition of *Actinidia chinensis* Planch. serum on gastric cancer and the effects of yam-containing serum on the chondrogenic differentiation have been reported [46, 48]. Serum pharmacology is a scientific analysis method for extracting serum-containing drugs from animals taken TCM orally [46, 49, 50].

The innovation of our article was to use TSW decoction to act on primary RAEC and study its promotion of angiogenesis and possible pathways. As far as we know, we were the first to demonstrate that the TSW-containing serum enhanced angiogenesis of RAEC by enhancing the RAEC viability and migration. LC-MS was used to analyze all the components of TSW decoction and combined with previous studies. We further studied its possible mechanism of action and blocked experiments with HIF-1*α* inhibitors, confirming that TSW decoction may partially promote endothelial cell angiogenesis through the VEGF/VHL/HIF-1*α* signaling pathway. In future research, we will further explore the action pathways of different components in TSW decoction. We will further study the mechanism of TSW decoction to develop effective drugs for the treatment of fracture patients.

## 5. Conclusion

Altogether, our results for the first time demonstrated that the TSW-containing serum enhanced angiogenesis of RAEC by enhancing the RAEC viability and migration. Mechanistic studies revealed that the effects of TSW-containing serum might be related to the VEGF/VHL/HIF-1*α* signaling pathway in the RAEC angiogenesis.

## Figures and Tables

**Table 1:** LC conditions.

Time (min)	Parameter
0	A: 2%; B: 98%
2	A: 4%; B: 96%
4	A: 10%; B: 90%
7	A: 14.5%; B: 85.5%
8	A: 14.5%; B: 85.5%
12	A: 23%; B: 77%
16	A: 46%; B: 54%
26	A: 100%; B: 0%
28	A: 100%; B: 0%
28.5	A: 2%; B: 98%

**Table 2:** Primer sequences.

Name	Forward primer	Reverse primer
HIF-1*α*	ACGTTCCTTCGATCAGTTGTCACC	GGCAGTGGTAGTGGTGGCATTAG
VHL	TTTGTGCCATCTCTCAATGTTG	GGCATCGCTCTTTCAGAGTATA
VEGF	ATCGAGTACATCTTCAAGCCAT	GTGAGGTTTGATCCGCATAATC
GAPDH	AGTCCACTGGCGTCTTCAC	GAGGCATTGCTGATGATCTTGA

## Data Availability

All the data generated or analyzed in this study are included in this manuscript, and the authors have confirmed the accuracy of the data.

## Abbreviations

EPO: Erythropoietin
HRG: Hypoxia responsive genes
HIF-1*α*: Hypoxia-inducible factor-1*α*
LC-MS: Liquid chromatography-mass spectrometry
RAEC: Rat aortic endothelial cells
SD: Sprague-Dawley
TCM: Traditional Chinese medicines
TSW: Taohong Siwu
VEGF: Vascular endothelial growth factor
VHL: Hippel-Lindau.

## Supplementary Materials

Supplementary Materials Figure S1: positive ion chromatogram and negative ion chromatogram of TSW Decoction were detected by LC-MS. Table S1: the components of TSW Decoction were detected by LC-MS.
