# Supplementary material for: Taohong Siwu-Containing Serum Enhances Angiogenesis in Rat Aortic Endothelial Cells by Regulating the VHL/HIF-1α/VEGF Signaling Pathway
Source: Evid Based Complement Alternat Med. 2021 Nov 22;2021:6610116. doi: 10.1155/2021/6610116 (PMC8629617; doi:10.1155/2021/6610116)
Supplement: Supplementary Materials — Figure S1: positive ion chromatogram and negative ion chromatogram of TSW Decoction were detected by LC-MS. Table S1: the components of TSW Decoction were detected by LC-MS. [file 6610116.f1.zip › 6610116.f1/Figure S1 (1).docx]

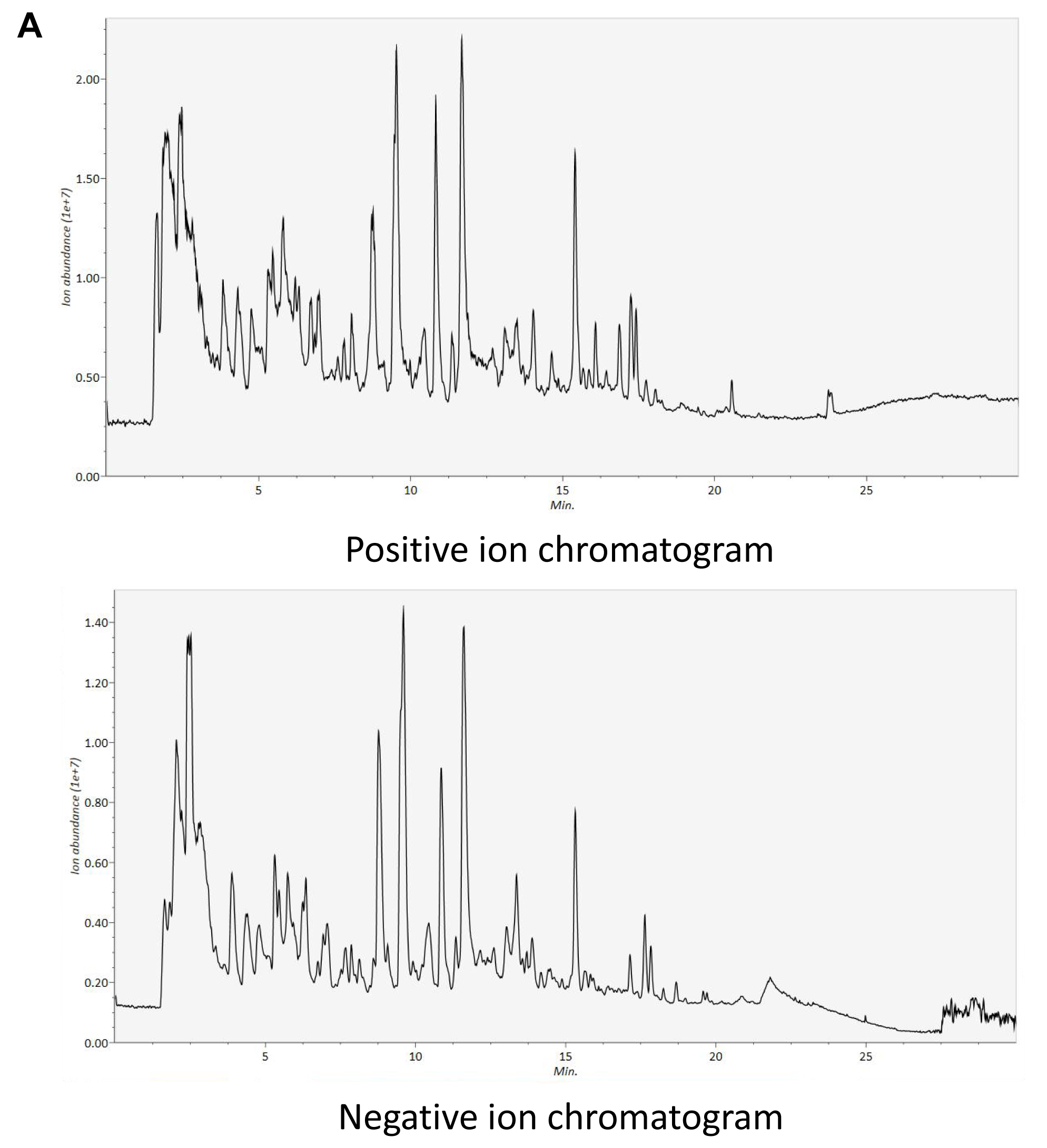


**Figure. S1 Positive ion chromatogram and negative ion chromatogram of TSW Decoction were detected by LC-MS.**
