# Supplementary material for: Taohong Siwu-Containing Serum Enhances Angiogenesis in Rat Aortic Endothelial Cells by Regulating the VHL/HIF-1α/VEGF Signaling Pathway
Source: Evid Based Complement Alternat Med. 2021 Nov 22;2021:6610116. doi: 10.1155/2021/6610116 (PMC8629617; doi:10.1155/2021/6610116)
Supplement: Supplementary Materials — Figure S1: positive ion chromatogram and negative ion chromatogram of TSW Decoction were detected by LC-MS. Table S1: the components of TSW Decoction were detected by LC-MS. [file 6610116.f1.zip › 6610116.f1/Revised table S1.pdf]

Alignment ID	Average Rt(min)	Metabolite name	Formula	Average Mz	Total score	Adduct type	Ms^2	Ontology
52	5.15	ETHYL METHYL KETONE	C4H8O	73.06543	100	[M+H]+	161210	Ketones
186	11.694	L-2,3-DIAMINOPROPIONIC ACID	C3H8N2O2	105.06596	100	[M+H]+	204032	L-alpha-amino acids
261	8.113	5,6-Dihydrouracil	C4H6N2O2	115.05071	100	[M+H]+	4993	Pyrimidones
492	11.687	3-Ureidopropionic acid	C4H8N2O3	133.06078	100	[M+H]+	335360	Ureas
515	5.899	Malate	C4H6O5	135.02771	100	[M+H]+	29312	Beta hydroxy acids and derivatives
800	12.299	Mevalonic Acid Lactone	C6H10O3	153.05099	100	[M+H]+	116684	Delta valerolactones
922	10.288	N-Methyllysine	C7H16N2O2	161.12932	100	[M+H]+	12917	L-alpha-amino acids
924	1.853	Alanopine	C6H11NO4	162.0705	100	[M+H]+	117495	Alanine and derivatives
932	13.843	7-hydroxy-coumarin	C9H6O3	163.03499	100	[M+H]+	49640	7-hydroxycoumarins
963	18.881	DL-5-HYDROXYLYSINE	C6H14N2O3	163.10887	100	[M+H]+	102186	Alpha amino acids
980	5.756	trans-p-Hydroxycinnamic acid	C9H8O3	165.05173	100	[M+H]+	179218	Hydroxycinnamic acids
1035	2.869	Quinolinic Acid	C7H5NO4	168.02499	100	[M+H]+	174653	Pyridinecarboxylic acids
1083	9.641	Lupinine	C10H19NO	170.05339	100	[M+H]+	11592	Lupinine-type alkaloids
1131	2.287	3-DEHYDROSHIKIMATE	C7H8O5	173.03955	100	[M+H]+	45494	Cyclohexenones
1292	19.485	Nicotinoylglycine	C8H8N2O3	181.06015	100	[M+H]+	13033	N-acyl-alpha amino acids
1337	19.694	Triethylphosphat	C6H15O4P	183.07817	100	[M+H]+	17393	Trialkyl phosphates
1459	10.295	N6-Acetyl-L-lysine	C8H16N2O3	189.12482	100	[M+H]+	40870	D-alpha-amino acids

1493	20.752	2,6-Diaminopimelic acid	C7H14N2O4	191.10136	100	[M+H]+	48135	Alpha amino acids
1711	19.074	Licarin A	C20H22O4	203.0988	100	[M+H]+	142406	2-arylbenzofuran flavonoids
1764	6.016	Isoeugenitol	C11H10O4	207.06793	100	[M+H]+	491	
1814	5.957	Nicotinoylcholine	C11H17N2O2	209.1275	100	[M+H]+	1666	Pyridinecarboxylic acids
2028	1.943	Theophylline	C7H8N4O2	219.02164	100	[M+H]+	3219624	Xanthines
2052	1.747	Sorbitol	C6H14O6	220.92906	100	[M+H]+	4827	Sugar alcohols
2087	5.99	Sinapinic acid	C11H12O5	225.08116	100	[M+H]+	3118	Hydroxycinnamic acids
2187	7.83	L-Prolyl-L-isoleucine	C11H20N2O3	229.14938	100	[M+H]+	266127	Dipeptides
2266	6.597	teasperin	C13H12O4	233.08417	100	[M+H]+	760	1-benzopyrans
2495	3.414	flavone	C15H10O2	245.05769	100	[M+H]+	99970	Flavones
2545	3.547	lenticin	C14H18N2O2	247.07271	100	[M+H]+	4846	Alpha amino acids
2613	12.074	2-hydroxyalantolactone	C15H20O3	249.10855	100	[M+H]+	387	Eudesmanolides, secoeudesmanolides, and derivatives
2643	4.712	Dihydrojasmonic Acid	C12H20O3	251.05487	100	[M+H]+	32135	Jasmonic acids
2979	1.695	Mianserin hydrochloride	C18H21ClN2	265.17084	100	[M+H]+	3812	Dibenzazepines
3007	6.765	resveratrol	C14H12O3	267.04297	100	[M+H]+	37483	Stilbenes
3009	3.338	aspernigrin A	C13H12N2O2	267.05438	100	[M+H]+	75460	Nicotinamides
3432	19.99	7,4'-DIMETHOXYISOFLAVONE	C17H14O4	283.18716	100	[M+H]+	18849	7-O-methylisoflavones
3737	19.858	Genistein	C15H10O5	293.16678	100	[M+H]+	38721	Isoflavones
3788	1.973	cyclopenin	C17H14N2O3	295.10757	100	[M+H]+	1870360	
4040	13.376	Nopaline	C11H20N4O6	305.14627	100	[M+H]+	1003	Arginine and derivatives
4255	2.126	Xanthinol	C13H21N5O4	312.16678	100	[M+H]+	36142	Xanthines
4703	19.121	Aleuretic Acid	C16H32O5	327.21405	100	[M+H]+	14462	
4742	15.023	Tulipinolide	C17H22O4	329.11618	100	[M+H]+	127352	Germacranolides and derivatives
4774	5.308	Paroxetine	C19H20FNO3	330.151	100	[M+H]+	16916	Phenylpiperidines

4962	7.04	Nicotinic acid mono nucleotide	C11H14NO9P	336.04834	100	[M+H]+	2576	Nicotinic acid nucleotides
5037	7.347	Griseofulvic Acid	C16H15ClO6	339.06458	100	[M+H]+	49005	
5040	7.777	rhodioloside	C14H20O7	339.09424	100	[M+H]+	13768	O-glycosyl compounds
5041	9.301	Heptelidic acid chlorohydrin	C15H21ClO5	339.09525	100	[M+H]+	50751	Terpene lactones
5212	3.215	Oxadiazone	:15H18Cl2N2O:	345.07617	100	[M+H]+	27825	Dichlorobenzenes
5285	16.862	Oryzalin	C12H18N4O6S	347.10187	100	[M+H]+	31464	Dinitroanilines
5534	5.302	Triacetyl resveratrol	C20H18O6	355.11752	100	[M+H]+	134725	Stilbene glycosides
5639	18.683	Lychnopholide	C20H22O6	359.14291	100	[M+H]+	35515	Terpene lactones
5654	17.193	Ochropposinine	C21H30N2O3	359.23285	100	[M+H]+	3191	Beta carbolines
5743	6.083	alpha-guaiaconic acid	C20H20O5	363.11969	100	[M+H]+	84156	2,5-diphenylfurans
6124	6.452	sesamin	C20H18O6	377.1004	100	[M+H]+	3652	Furanoid lignans
6222	10.359	Atalaphylline	C23H25NO4	380.18741	100	[M+H]+	55090	
6380	5.615	Uncarine c	C21H24N2O4	386.20609	100	[M+H]+	1114	Indolizidines
6447	20.628	dehydroindicolac tone	C21H18O6	389.09485	100	[M+H]+	143	Psoralens
6501	5.457	Curcumin	C21H20O6	391.11505	100	[M+H]+	1088	Curcuminoids
6506	16.719	candidone	C22H24O4	391.13052	100	[M+H]+	42039	8-prenylated flavanones
6646	17.767	ROTENONE	C23H22O6	395.19907	100	[M+H]+	49020	Rotenones
6724	4.461	ROBUSTIC ACID	C22H20O6	398.15839	100	[M+H]+	81289	Hydroxyisoflavonoid s
6771	8.306	Conopharyngine	C23H30N2O4	399.2287	100	[M+H]+	280	Ibogan-type alkaloids
7082	18.915	CHOLESTEROL	C27H46O	409.34387	100	[M+H]+	86	Cholesterols and derivatives
7589	2.294	tephrosin	C23H22O7	428.17053	100	[M+H]+	417297	Rotenones
8456	2.198	Himeic acid A	C22H29NO8	458.1785	100	[M+H]+	420810	Long-chain fatty acids
8481	11.245	Prednisolone_Teb utate	C27H38O6	459.27338	100	[M+H]+	3273	Gluco/mineralocorti coids, progestogins and derivatives
8745	17.121	Hecogenin	C27H42O4	469.27231	100	[M+H]+	32490	Triterpenoids

8985	11.551	Flavone base + 2O, 2MeO, C-	C23H24O11	477.13791	100	[M+H]+	42294	Flavone C-glycosides
9310	3.63	Aquayamycin	C25H26O10	487.15994	100	[M+H]+	1728	Angucyclines
9320	4.81	Rhizocarpic Acid	C28H23NO6	487.1853	100	[M+H]+	89565	Phenylalanine and derivatives
9567	12.508	Deoxypumiloside	C26H28N2O8	497.19696	100	[M+H]+	76522	Carbolines
9790	8.711	hypericin	C30H16O8	505.14661	100	[M+H]+	135393	Benzopyrenes
10203	7.802	Dendroamide B	C21H24N6O4S3	521.10931	100	[M+H]+	250088	Macrolactams
11453	13.434	Flavanone base + 4O, O-Hex, C-Pen	C26H30O15	583.16516	100	[M+H]+	6409	Flavanone C,O-glycosides
11677	6.037	Quercetin-3-O-vicianoside	C26H28O16	597.14557	100	[M+H]+	23637	Flavonol O-glycosides
11692	13.019	Astaxanthin	C40H52O4	597.39447	100	[M+H]+	77	Xanthophylls
12040	16.896	Peltatoside	C26H28O16	619.12762	100	[M+H]+	4970	Flavonoid-3-O-glycosides
12805	13.174	Flavone base + 3O, 2MeO, O-Hex, O-HexA	C29H32O18	669.16595	100	[M+H]+	26820	Flavone O-glycosides
12986	2.849	Flavone base + 3O, 2MeO, O-HexA-HexA	C29H30O19	683.14996	100	[M+H]+	383	Flavone O-glycosides
14545	16.453	Soyasapogenol B base + O-HexA+HexA+dHex	C48H74O19	955.48279	100	[M+H]+	26	Triterpene saponins
76	2.218	Glyceric acid	C3H6O4	105.01961	100	[M-H]-	131691	Sugar acids and derivatives
351	8.227	2-HYDROXYPHENYLACETIC ACID	C8H8O3	151.03952	100	[M-H]-	34748	
423	7.781	Acesulfame	C4H5NO4S	161.98697	100	[M-H]-	15	Organic sulfuric acids and derivatives
464	19.478	raspberry ketone	C10H14O2	165.09285	100	[M-H]-	10965	1-hydroxy-2-unsubstituted benzenoids
537	5.665	Ascorbic acid	C6H8O6	175.02539	100	[M-H]-	1596	Butenolides

622	8.169	METHYL VANILLATE	C8H8O4	181.05023	100	[M-H]-	29830	M-methoxybenzoic acids and derivatives
624	7.446	Evernininic Acid	C9H10O4	181.05173	100	[M-H]-	1745	
636	4.62	Carbamimidothioic acid	C3H8N2O3S2	182.9897	100	[M-H]-	4954	Organosulfonic acids
698	12.284	Hydroxysuberic acid	C8H14O5	189.07722	100	[M-H]-	14110	Organic acids
737	21.817	N-Methyltyrosine	C10H13NO3	194.08344	100	[M-H]-	37291	Tyrosine and derivatives
843	15.505	PYROGALLIN	C11H8O4	203.03394	100	[M-H]-	9853	Tropolones
899	11.752	Methyl Haematommate	C10H10O5	209.0439	100	[M-H]-	64973	
907	14.816	Divaricatinic acid	C11H14O4	209.08247	100	[M-H]-	11996	
916	10.204	Methoxytyrosine	C10H13NO4	210.07693	100	[M-H]-	96882	Amino acids
960	17.771	5-Methoxypsoralen	C12H8O4	215.03392	100	[M-H]-	4269	5-methoxypsoralens
1002	7.09	Pantothenic acid	C9H17NO5	218.10403	100	[M-H]-	141201	Secondary alcohols
1018	6.616	5-Hydroxytryptophan	C11H12N2O3	219.07727	100	[M-H]-	137	Serotonins
1199	17.281	austadiol	C12H12O5	235.06226	100	[M-H]-	122772	Azaphilones
1343	13.502	Indole-3-acetyl-L-alanine	C13H14N2O3	245.0932	100	[M-H]-	1390	Amino acids
1513	10.157	Glucosamine-6-phosphate	C6H14NO8P	258.03976	100	[M-H]-	9937	Hexose phosphates
1524	10.157	Peucenin	C15H16O4	259.07434	100	[M-H]-	117149	Chromones
1610	22.25	FA 16:3+1O	C16H26O3	265.17984	100	[M-H]-	12895	Oxidized fatty acids
1738	18.942	alpha,beta-Dihydroresveratrol	C14H14O3	275.09152	100	[M-H]-	6201	Stilbene glycosides
1852	28.219	Oleic acid	C18H34O2	281.24857	100	[M-H]-	9454	Long-chain fatty acids
1963	18.942	Epicatechin	C15H14O6	289.07214	100	[M-H]-	10057	Catechins
2025	7.767	Glutamylphenylalanine	C14H18N2O5	293.12347	100	[M-H]-	17208	Amino acids
2106	4.643	picein	C14H18O7	297.12094	100	[M-H]-	39467	Phenolic glycosides

2111	8.335	Nialamide	C16H18N4O2	297.13547	100	[M-H]-	18378	Pyridinecarboxylic acids and derivatives
2428	21.875	Octadecanedioic acid	C18H34O4	313.23846	100	[M-H]-	15048	Long-chain fatty acids
2517	15.937	Hydroxyhaemoventosine	C15H12O8	319.04086	100	[M-H]-	209	
2531	18.622	barceloneic acid A	C16H16O7	319.12064	100	[M-H]-	15222	Diphenylethers
2650	9.151	Coumaroyl Hexoside (isomer of 690, 691)	C15H18O8	325.09415	100	[M-H]-	235511	Coumaric acid and derivatives
2662	18.838	FA 18:3+3O	C18H30O5	325.20096	100	[M-H]-	19970	Oxidized fatty acids
2688	9.858	Dihydrocoumaroyl Hexoside	C15H20O8	327.10574	100	[M-H]-	12064	Coumaric acid and derivatives
2718	5.642	Adenosine 3',5'-cyclic monophosphate	C10H12N5O6P	328.04575	100	[M-H]-	4192	3',5'-cyclic purine nucleotides
2764	19.771	FA 18:1+3O	C18H34O5	329.23309	100	[M-H]-	58692	Oxidized fatty acids
2788	3.524	Gallic acid hexoside	C13H16O10	331.06577	100	[M-H]-	2395	Phenolic glycosides
3010	1.895	MGMG 2:0	C11H20O9	341.10788	100	[M-H]-	178420	Lipids
3048	16.034	Isousnic acid	C18H16O7	343.07962	100	[M-H]-	1319	
3109	23.056	Gibberellic acid	C19H22O6	345.13431	100	[M-H]-	14678	C19-gibberellin 6-carboxylic acids
3225	9.354	Leprapinic acid	C20H16O6	351.08273	100	[M-H]-	15544	
3236	21.225	Isoflavone base + 2O + 1MeO + 1Prenyl	C21H20O5	351.12286	100	[M-H]-	52	Prenylated isoflavones
3271	5.197	6:3+6O fatty acyl hexoside	C12H18O12	353.07211	100	[M-H]-	2810	Fatty acyl hexosides
3282	8.694	Caffeoylquinic acid	C16H18O9	353.08948	100	[M-H]-	21247	Quinic acids and derivatives
3341	9.997	Feruloyl Hexoside (isomer of 849)	C16H20O9	355.103	100	[M-H]-	2803	Ferulic acid and derivatives
3827	12.938	loganic acid	C16H24O10	375.1297	100	[M-H]-	141205	
4145	8.156	Geniposide	C17H24O10	387.12961	100	[M-H]-	4434	Iridoid glycosides
4256	16.956	Dehydrorotenone	C23H20O6	391.21652	100	[M-H]-	40965	Rotenones

4359	13.693	Hexose + C10H19O2	C16H30O8	395.19009	100	[M-H]-	17597	Terpene glycosides
4382	5.819	Clavulanic acid	C8H9NO5	397.08978	100	[M-H]-	44039	
4455	18.494	Hexose + C13H17O3	C19H28O9	399.16599	100	[M-H]-	16911	Terpene glycosides
4481	16.208	Cetraric acid	C20H18O9	401.07465	100	[M-H]-	27582	
4493	8.117	Gentiopicroside	C16H20O9	401.10895	100	[M-H]-	4267	Glycosyl
5076	3.701	Glucoiberin	C11H21NO10S3	422.02661	100	[M-H]-	92	Alkylglucosinolates
5323	12.442	Cosmosiin	C21H20O10	431.09763	100	[M-H]-	1508	Flavone O-glycosides
5383	12.034	Flavanone + 3O, O-Hex	C21H22O10	433.11215	100	[M-H]-	12615	Flavanone O-glycosides
5719	18.376	Flavone base + 2O, 1MeO, C-	C22H22O10	445.11581	100	[M-H]-	15702	Flavone C-glycosides
6025	2.605	Deoxyuridine	C9H12N2O5	455.09995	100	[M-H]-	589	Pyrimidine 2'-deoxyribonucleosides
6251	10.521	Isoquercitrin	C21H20O12	463.08081	100	[M-H]-	407	Flavonoid-3-O-glycosides
6279	19.717	Rubranoside B	C24H32O9	463.19543	100	[M-H]-	17835	
6443	6.834	Fumarprotocetraric Acid	C22H16O12	470.92657	100	[M-H]-	1420	Depsides and depsidones
6992	18.683	Biochanin-7-O-glucoside	C22H22O10	491.11945	100	[M-H]-	89740	Isoflavone O-glycosides
7061	9.02	methoxy-myricetin-3-O-hexoside	C22H22O13	493.09167	100	[M-H]-	18677	Flavonoid-3-O-glycosides
8187	18.25	Flavone base + 3O, C-Pen, C-	C25H26O13	533.12787	100	[M-H]-	128391	Flavone C,C-glycosides
8951	9.091	Flavone base + 3O, C-Hex, C-	C26H28O14	563.14038	100	[M-H]-	40369	Flavone C,C-glycosides
9055	24.242	LPC 18:1	C26H52NO7P	566.3465	100	[M-H]-	6354	Lipids
9585	17.055	Disinapoyl Hexoside	C28H32O14	591.17072	100	[M-H]-	14963	Sinapinic acid and derivatives
9924	8.59	kaempferol-3-O-hexoxyl(1-6)hexoside	C27H30O16	609.1449	100	[M-H]-	120457	Flavonoid-3-O-glycosides
10354	6.076	Rhamnetin	C16H12O7	631.10876	100	[M-H]-	1035	Flavonols

10511	10.571	isosakuranetin-7-O-neohesperidoside	C28H34O14	639.19434	100	[M-H]-	38686	Flavonol O-glycosides
11613	12.362	Tetrasaccharides (Hex-Hex-Hex-Hex)	C24H42O21	711.2168	100	[M-H]-	323	Sugars
11785	15.974	Coumarin base + 1O, 1MeO, O-(C26H31O12)	C36H38O16	725.20746	100	[M-H]-	808	Coumarin and derivatives
12761	19.456	Oleanane -4H, + 2O, 1COOH, O-HexA, HexA	C42H62O16	821.39996	100	[M-H]-	64	Triterpene saponins
13589	19.787	Soyasapogenol B base + O-HexA-Pen-dHex	C47H76O17	911.50415	100	[M-H]-	11	Triterpene saponins
13847	16.255	Isorhamnetin-3-O-glucoside	C22H22O12	955.21661	100	[M-H]-	9244	Flavonoid-3-O-glycosides
13852	19.463	Soyasapogenol B base + O-HexA-HexA-dHex	C48H76O19	955.48981	100	[M-H]-	26	Triterpene saponins
14041	19.524	Soyasaponin Bb	C48H78O18	987.51886	100	[M-H]-	5	Triterpene saponins
857	7.064	N-Methylbenzylamine Hydrochloride	C8H12ClN	158.07584	99.9	[M+H]+	5210	Phenylmethylamines
937	13.098	Umbelliferone	C9H6O3	163.03629	99.9	[M+H]+	4711	
960	1.724	delta-Hydroxylysine	C6H14N2O3	163.10501	99.9	[M+H]+	12758	Alpha amino acids
1033	18.598	Dihydrojasmone	C11H18O	167.14093	99.9	[M+H]+	23184	Cyclic ketones
1187	9.317	Citrulline	C6H13N3O3	176.02702	99.9	[M+H]+	10992	L-alpha-amino acids
1238	2.017	Cysteinylglycine	C5H10N2O3S	179.05014	99.9	[M+H]+	186956	Dipeptides
1609	15.42	Xanthopterin	C6H5N5O2	197.07614	99.9	[M+H]+	1444663	Pterins and derivatives
2130	18.69	METYRAPONE	C14H14N2O	227.11771	99.9	[M+H]+	9174	
2237	22.332	Eremanthin	C15H18O2	231.13081	99.9	[M+H]+	13844	Guaianolides and derivatives
2603	6.925	xanthocerin	C13H12O5	249.07941	99.9	[M+H]+	35126	1-benzopyrans

2825	15.476	1-Methylpseudouridine	C10H14N2O6	259.09052	99.9	[M+H]+	1049	Nucleoside and nucleotide analogues
3071	18.116	N-acetyltryptophan	C13H14N2O3	269.11234	99.9	[M+H]+	19433	N-acyl-alpha amino acids
3306	22.952	L-Saccharopine	C11H20N2O6	277.13736	99.9	[M+H]+	15934	Glutamic acid and derivatives
3308	6.072	3,7-Epoxycaryophyllan-6-Ol	C15H26O2	277.14337	99.9	[M+H]+	29026	Oxepanes
3451	22.408	9,10-alpha-Epoxy-eremanthin	C15H18O3	284.32516	99.9	[M+H]+	60981	Gamma butyrolactones
3470	14.293	Articaine HCl	C13H20N2O3S	285.12445	99.9	[M+H]+	458	Thiophene carboxylic acids and derivatives
4075	9.123	Epigallocatechin	C15H14O7	307.0722	99.9	[M+H]+	30097	Epigallocatechins
5168	7.612	2-alpha-Mannobiose	C12H22O11	343.12131	99.9	[M+H]+	42948	O-glycosyl compounds
6391	19.288	10-Hydroxycamptothecin	C20H16N2O5	387.09772	99.9	[M+H]+	8255	Camptothecins
6468	13.441	Hexose + C13H21O2	C19H32O8	389.21332	99.9	[M+H]+	14606	Fatty acyl hexosides
7290	2.235	Coralyne Chloride	C22H22ClNO4	417.15936	99.9	[M+H]+	17817	Isoquinolines and derivatives
8151	1.876	Archangelicine	C24H26O7	449.06317	99.9	[M+H]+	4690	Angular furanocoumarins
8444	18.596	Vitamin K1	C31H46O2	457.36362	99.9	[M+H]+	49	Vitamin K compounds
10552	12.018	Vicriviroc Malate	C32H44F3N5O[illegible]	534.30304	99.9	[M+H]+	348	Trifluoromethylbenzenes
10875	5.749	Flavone base + 3O, 1MeO, O-MalonylHex	C25H24O14	549.12781	99.9	[M+H]+	52828	Flavone O-glycosides
12628	18.258	Flavonol base + 4O, 1MeO, O-Hex-Hex	C28H32O18	657.16937	99.9	[M+H]+	6291	Flavonol O-glycosides

12971	6.501	Chondramide B	C36H45ClN4O7	681.30804	99.9	[M+H]+	536	Hybrid peptides
13071	5.15	AERUGINOSIN 98A	C29H45ClN6O9[illegible]	689.27386	99.9	[M+H]+	13816	
13913	10.104	echinacoside	C35H46O20	809.24078	99.9	[M+H]+	456340	Oligosaccharides
13939	18.637	Salinomycin, Sodium	C42H69NaO11	811.43372	99.9	[M+H]+	16	Diterpene glycosides
904	2.625	1,3,7-Trimethyluric acid	C8H10N4O3	209.06567	99.9	[M-H]-	10562	Xanthines
911	19.764	Jasmonic acid	C12H18O3	209.12071	99.9	[M-H]-	10401	Jasmonic acids
1550	3.592	Asp-Glu	C9H14N2O7	261.0744	99.9	[M-H]-	36559	Dipeptides
1915	8.849	Piperanine	C17H21NO3	286.14246	99.9	[M-H]-	0	Benzodioxoles
2333	10.784	Glutamyltyrosine	C14H18N2O6	309.10638	99.9	[M-H]-	1109	Amino acids
2825	9.215	Chiodectonic acid	C15H10O9	333.0177	99.9	[M-H]-	42457	
3099	20.19	Obtusatic acid	C18H18O7	345.09372	99.9	[M-H]-	32661	
3448	13.932	Deoxyloganic acid	C16H24O9	359.13092	99.9	[M-H]-	28276	Iridoid glycosides
3513	5.634	Guanosine-5'-monophosphate	C10H14N5O8P	362.05264	99.9	[M-H]-	17521	Purine ribonucleoside monophosphates
3818	21.517	Pseudoplacodiolic acid	C19H20O8	375.10306	99.9	[M-H]-	12901	
4128	15.922	Salazinic acid	C18H12O10	387.02615	99.9	[M-H]-	86	
4147	19.839	Divaricatic acid	C21H24O7	387.14267	99.9	[M-H]-	18705	
4642	18.838	Ginkgolide A	C20H24O9	407.13226	99.9	[M-H]-	16917	Ginkgolides and bilobalides
5078	9.959	Gluconasturtiin	C15H21NO9S2	422.06073	99.9	[M-H]-	125	Alkylglucosinolates
5284	17.694	Anziaic acid	C24H30O7	429.18396	99.9	[M-H]-	942	
5397	10.068	Geniposide (Not validated)	C17H24O10	433.13763	99.9	[M-H]-	87961	Iridoid glycosides
5466	9.097	Artocaprin	C26H28O6	435.17902	99.9	[M-H]-	296	3-prenylated flavones
5665	3.06	Halometasone	C22H27ClF2O5	443.14175	99.9	[M-H]-	43793	21-hydroxysteroids
6063	1.741	Smilagenin Acetate	C29H46O4	456.85809	99.9	[M-H]-	66950	
6740	20.408	Umbilicaric acid	C25H22O10	481.10562	99.9	[M-H]-	11102	
9228	5.693	Flavone base + 4O, C-(dehydro-dHex)-dHex	C27H28O14	575.13666	99.9	[M-H]-	50829	Flavone C-glycosides

9342	14.05	S(8-8)S hexoside	C28H36O13	579.21136	99.9	[M-H]-	30936	Lignols
9499	14.306	Etoposide	C29H32O13	587.17432	99.9	[M-H]-	10435	Lignols
9922	13.296	Flavonol base + 4O, O-dHex-Hex	C27H30O16	609.14191	99.9	[M-H]-	1038	Flavonol O-glycosides
10866	19.585	Medicagenic acid base -H2O + O-HexA	C36H54O11	661.36084	99.9	[M-H]-	1218	Triterpene saponins
12655	19.847	Soyasapogenol B base + O-HexA-HexA	C42H66O15	809.43811	99.9	[M-H]-	41	Triterpene saponins
297	2.603	Valine	C5H11NO2	118.08325	99.8	[M+H]+	2539	Valine and derivatives
430	1.924	Pyroglutamic acid	C5H7NO3	130.04642	99.8	[M+H]+	1033676	Alpha amino acids and derivatives
509	6.953	INDOXYL SULFATE	C8H7NO4S	134.05721	99.8	[M+H]+	221108	Arylsulfates
869	19.369	hyocholic acid	C24H40O5	158.15073	99.8	[M+H]+	62161	
955	12.112	Methyl cinnamate	C10H10O2	163.07108	99.8	[M+H]+	94306	Cinnamic acid and derivatives
1274	8.619	Meglutol	C6H10O5	180.08383	99.8	[M+H]+	15223	Hydroxy fatty acids
1521	1.876	PYROCATECHUIC ACID	C7H6O4	192.99326	99.8	[M+H]+	18547	Salicylic acids
1579	6.902	MENTHOL(-)	C10H20O	195.11775	99.8	[M+H]+	17816	Menthane monoterpenoids
1881	5.199	PHOSPHOCREATINE	C4H10N3O5P	212.04619	99.8	[M+H]+	69416	Alpha amino acids and derivatives
2217	16.22	D-(?)-Quinic acid	C7H12O6	231.05534	99.8	[M+H]+	1449	Quinic acids and derivatives
2372	13.886	Ketamine	C13H16ClNO	238.10249	99.8	[M+H]+	9814	Chlorobenzenes
3639	5.05	Arbutin	C12H16O7	290.1203	99.8	[M+H]+	5566	Phenolic glycosides
3779	7.983	Laurinterol	C15H19BrO	295.07211	99.8	[M+H]+	1183	Sesquiterpenoids
4563	18.991	phellopterin	C17H16O5	323.08563	99.8	[M+H]+	18158	5-methoxypsoralens
5330	22.439	Inosine-5'-monophosphate	C10H13N4O8P	349.05734	99.8	[M+H]+	10525	Purine ribonucleoside monophosphates
6224	12.977	pipernonaline	C21H27NO3	380.21201	99.8	[M+H]+	13558	Benzodioxoles

8961	12.603	Sibiromycin hemiaminal	C24H33N3O7	476.24197	99.8	[M+H]+	149501	Aminoglycosides
9383	6.327	Deoxygedunin	C28H34O6	489.22083	99.8	[M+H]+	46742	Naphthopyrans
11216	15.81	quercetin-3-O-pentosyl-pentoside	C25H26O15	567.13959	99.8	[M+H]+	499	Flavonoid-3-O-glycosides
11613	12.219	Flavonol base + 3O, O-Hex+C6H9O4	C27H28O15	593.15564	99.8	[M+H]+	93353	Flavonol O-glycosides
12269	5.661	eriocitrin	C27H32O15	635.13312	99.8	[M+H]+	99641	Flavonoid-7-O-glycosides
12372	10.242	S4:16(P3:14/F1:2)	C28H46O15	640.32422	99.8	[M+H]+	192	Saccharolipids
13121	5.246	Deoxyguanylic acid	C10H14N5O7P	695.09338	99.8	[M+H]+	448	Purine 2'-deoxyribonucleoside monophosphates
1958	5.602	CIANIDANOL	C15H14O6	289.06894	99.8	[M-H]-	3406	
2665	7.544	Aristolochic acid C	C16H9NO7	326.02747	99.8	[M-H]-	101	Alkaloids
3466	5.853	Indole-3-acetyl-L-tryptophan	C21H19N3O3	360.13214	99.8	[M-H]-	40715	Amino acids
4012	2.204	S-Adenosyl-homocysteine	C14H20N6O5S	383.11774	99.8	[M-H]-	389119	5'-deoxy-5'-thionucleosides
5180	5.365	Adenosine-5'-phosphosulfate sodium salt	:10H14N5O10P	426.01538	99.8	[M-H]-	568	Purine ribonucleoside monophosphates
5402	11.604	Mundulone	C26H26O6	433.16251	99.8	[M-H]-	1101	6-prenylated isoflavanones
6861	9.518	3-Hydroxyphysodic acid	C26H30O9	485.17889	99.8	[M-H]-	198	Depsides and depsidones
7086	17.043	Pentose-Hexose + C10H17	C21H36O10	493.23254	99.8	[M-H]-	763	Terpene glycosides
8020	11.599	Phosphatidylcholine lyso 16	C24H50NO7P	526.34802	99.8	[M-H]-	9373	2-acyl-sn-glycero-3-phosphocholines
10218	6.965	Oregonoyl A	C33H36O12	623.2179	99.8	[M-H]-	31055	
10447	12.914	3-O-vanilloyl ceanothic acid	C38H52O8	635.35535	99.8	[M-H]-	2378	Triterpenoids
326	4.535	Tyramine	C8H11NO	121.06094	99.7	[M+H]+	113270	Amino acids

613	5.74	cis,cis-Muconic acid	C6H6O4	143.02974	99.7	[M+H]+	68251	Medium-chain fatty acids
894	2.105	N-Isovalerylglycine	C7H13NO3	160.09268	99.7	[M+H]+	112935	N-acyl-alpha amino acids
982	2	1,5-Anhydrosorbitol	C6H12O5	165.07191	99.7	[M+H]+	363038	Monosaccharides
1657	7.116	methyl orsellinate	C9H10O4	200.08817	99.7	[M+H]+	45777	p-Hydroxybenzoic acid alkyl esters
1916	3.661	Prolylproline	C10H16N2O3	213.12076	99.7	[M+H]+	3828	Dipeptides
2023	7.756	Propionylcarnitin	C10H19NO4	218.13499	99.7	[M+H]+	49794	Acyl carnitines
2041	7.068	Meprobamate	C9H18N2O4	219.13028	99.7	[M+H]+	110197	Carbamate esters
2254	12.232	Amidopyrine	C13H17N3O	232.14029	99.7	[M+H]+	16232	Phenylpyrazoles
2389	17.671	Primuletin	C15H10O3	239.11996	99.7	[M+H]+	22501	Flavones
2606	17.238	Glu-Thr	C9H16N2O6	249.10455	99.7	[M+H]+	89305	Dipeptides
2994	7.372	Thiamine	C12H17N4OS]-	266.11032	99.7	[M+H]+	35076	Thiamines
4073	16.725	4'-Methylgenistein (Biochanin A)	C16H12O5	307.05215	99.7	[M+H]+	168	4'-O-methylisoflavones
9321	10.95	6-HYDROXYANGOLENSIC ACID METHYL ESTER	C27H34O8	487.20306	99.7	[M+H]+	38209	Limonoids
9355	19.672	Beauverolide I	C27H41N3O5	488.31564	99.7	[M+H]+	33582	Cyclic depsipeptides
9494	17.431	steganacin	C24H24O9	495.12097	99.7	[M+H]+	835	Lignan lactones
10001	8.09	BiakamideA,B	:26H42ClN3O3:	512.27509	99.7	[M+H]+	49	
10415	2.135	Rolitetracycline	C27H33N3O8	528.22968	99.7	[M+H]+	4875	Tetracyclines
11444	5.934	Streptomycin	C21H39N7O12	582.27747	99.7	[M+H]+	1029	Aminocyclitol glycosides
14006	19.726	Ammonium Glycyrrhizinate (AMGZ)	C42H65NO16	823.40424	99.7	[M+H]+	103	Triterpene saponins
14388	8.584	Beta-Methoxy-Asn-Albicidin	C45H42N6O14	891.27942	99.7	[M+H]+	31593	Benzanilides
14400	4.421	betamethoxyvaleroyl CoA	28H46N7O18P3	894.19739	99.7	[M+H]+	112	3-oxo-acyl CoAs
3806	4.686	Alectorialic acid	C18H16O9	375.06644	99.7	[M-H]-	57525	

4170	1.667	5-PHOSPHO-D-RIBOSE 1-DIPHOSPHATE	C5H13O14P3	388.94003	99.7	[M-H]-	177015	Pentose phosphates
4619	16.255	3-Methylthiopropyl-Glucosinolate	C11H21NO9S3	406.03467	99.7	[M-H]-	19	Alkylglucosinolates
8461	3.714	Strictosamide	C26H30N2O8	543.19305	99.7	[M-H]-	27443	Carbolines
9318	8.893	Uridine diphosphate glucuronic acid	:15H22N2O18P	579.03119	99.7	[M-H]-	28264	Pyrimidine nucleotide sugars
12927	14.477	Phosphatidylcholine 16	C46H80NO8P	836.58746	99.7	[M-H]-	250085	Phosphatidylcholines
455	3.771	CITRACONIC ACID	C5H6O4	131.02953	99.6	[M+H]+	67788	Methyl-branched fatty acids
1448	15.314	Azelaic acid	C9H16O4	189.10738	99.6	[M+H]+	31564	Medium-chain fatty acids
2455	5.99	beta-lapachone	C15H14O3	243.09271	99.6	[M+H]+	2092	Naphthopyranones
3274	2.247	gamma-Glutamylglutamine	C10H17N3O6	276.11456	99.6	[M+H]+	238426	Dipeptides
4271	13.588	aflatoxin B1	C17H12O6	313.07559	99.6	[M+H]+	1911	Difurocoumarocyclopentenones
6695	18.178	swertiamarin	C16H22O10	397.11874	99.6	[M+H]+	31881	O-glycosyl compounds
8335	23.124	LPE 16:0	C21H44NO7P	454.28809	99.6	[M+H]+	8693	Lipids
10233	10.584	Melezitose	C18H32O16	522.2077	99.6	[M+H]+	54368	Oligosaccharides
10689	13.899	4-N-methyllyaloside	C28H33N2O9	541.21283	99.6	[M+H]+	1030	Terpene glycosides
5376	6.419	quercetin-3-O-pentoside	C20H18O11	433.06766	99.6	[M-H]-	121030	Flavonoid-3-O-glycosides
5611	8.709	Picrolichenic acid	C25H30O7	441.19162	99.6	[M-H]-	15006	
6321	22.258	agnuside	C22H26O11	465.19193	99.6	[M-H]-	20235	Iridoid O-glycosides
8723	6.555	N-Fructosyl S-(2-carboxypropyl)glutathione	:20H33N3O13!	554.17236	99.6	[M-H]-	32584	N-Fructosyl peptides
9812	12.956	Phosphatidylcholine lyso 22	C30H54NO7P	602.38794	99.6	[M-H]-	52079	2-acyl-sn-glycero-3-phosphocholines

10182	28.105	Ginsenoside compound K	C36H62O8	621.4425	99.6	[M-H]-	16129	Triterpenoids
250	5.13	1,4-Cyclohexanedione	C6H8O2	113.05463	99.5	[M+H]+	90353	Cyclic ketones
1731	13.673	Indole-3-pyruvic acid	C11H9NO3	204.06038	99.5	[M+H]+	2169	Indolyl carboxylic acids and derivatives
2522	9.123	N-L-Leucyl-L-leucine	C12H24N2O3	245.18103	99.5	[M+H]+	43084	Peptides
2870	5.961	Isoleucylglutamate	C11H20N2O5	261.13947	99.5	[M+H]+	57882	Dipeptides
4831	18.574	albocycline	C18H28O4	331.1832	99.5	[M+H]+	21385	Macrolides and analogues
5192	9.101	N-Fructosyl tyrosine	C15H21NO8	344.12796	99.5	[M+H]+	166196	N-Fructosyl amino acids
6225	13.733	12'-Apoastaxanthinal	C25H32O3	380.23953	99.5	[M+H]+	87546	Diterpenoids
8964	23.11	LPE 18:3	C23H42NO7P	476.27045	99.5	[M+H]+	5651	Lipids
9300	15.426	Enhydrin	C23H28O10	487.10999	99.5	[M+H]+	31508	Germacranolides and derivatives
12153	5.864	myricetin-3-rutinoside	C27H30O17	627.16547	99.5	[M+H]+	12406	Flavonoid-3-O-glycosides
12980	13.282	S4:19(P3:17/F1:2)	C31H52O15	682.36511	99.5	[M+H]+	589	Saccharolipids
13701	6.867	Flavone base + 3O, 2MeO, O-MalonylHex, O-guaiacylglycerol	C36H38O19	775.21716	99.5	[M+H]+	27136	Lignols
2129	8.766	Emodic acid	C15H8O7	299.01724	99.5	[M-H]-	297	
5517	13.485	5-O-methyllicoricidin	C27H34O5	437.23846	99.5	[M-H]-	53711	7-O-methylated isoflavonoids
6818	10.784	Dihydrogedunin	C28H36O7	483.24359	99.5	[M-H]-	152	Limonoids
8946	6.987	Theaflavin	C29H24O12	563.11371	99.5	[M-H]-	15652	Catechins
616	5.919	Ectoine	C6H10N2O2	143.0764	99.4	[M+H]+	2054	
1100	3.757	Gallic acid	C7H6O5	171.02263	99.4	[M+H]+	184500	Gallic acids
1435	10.049	Xylose	C5H10O5	189.01053	99.4	[M+H]+	71964	Pentoses
1533	3.066	Quinic acid	C7H12O6	193.06462	99.4	[M+H]+	28022	Quinic acids and derivatives

2282	7.315	Threonylleucine	C10H20N2O4	233.14319	99.4	[M+H]+	69462	Dipeptides
4189	28.137	Smenospongidine	C29H37NO3	310.30566	99.4	[M+H]+	27882	
5147	7.715	Caseadine	C20H23NO4	342.19437	99.4	[M+H]+	17327	Protoberberine alkaloids and derivatives
6453	5.654	Hexose + C11H12O4	C17H24O10	389.13849	99.4	[M+H]+	41656	Terpene glycosides
8582	14.171	FOLIC ACID	C19H19N7O6	464.13419	99.4	[M+H]+	45439	Glutamic acid and derivatives
11348	8.946	Dihydrocelastryl Diacetate	C33H44O6	575.237	99.4	[M+H]+	73931	Phenanthrenes and derivatives
14556	15.407	VINCRISTINE SULFATE	:46H58N4O14!	961.32458	99.4	[M+H]+	70888	Vinca alkaloids
2324	2.106	N-Acetylneuraminic acid	C11H19NO9	308.10428	99.4	[M-H]-	59338	N-acylneuraminic acids
5238	19.689	SANGUINARINE SULFATE	C20H15NO8S	428.05817	99.4	[M-H]-	12653	Quaternary benzophenanthridine alkaloids
6095	12.61	3-Hydroxycolensoic acid	C25H30O8	457.18759	99.4	[M-H]-	29951	Depsides and depsidones
6604	17.021	Speciosine	C28H31NO6	476.21338	99.4	[M-H]-	1910	Alkaloids
284	2.167	Glycine-Betaine	C5H11NO2	117.07249	99.3	[M+H]+	598931	Alpha amino acids
8990	13.554	Mundulone Acetate	C28H28O7	477.18469	99.3	[M+H]+	36659	6-prenylated isoflavanones
10398	16.681	MADECASSIC ACID	C30H48O6	527.32825	99.3	[M+H]+	283	Triterpenoids
14195	7.376	euphodendroidin S	C44H52N2O15	849.35443	99.3	[M+H]+	140	NA
1159	3.788	sorbicillin	C14H16O3	231.05363	99.3	[M-H]-	43639	Xylenols
1480	11.687	Purpurin	C14H8O5	255.02399	99.3	[M-H]-	269	Hydroxyanthraquinones
4519	28.155	4-Methylpentyl glucosinolate	C13H25NO9S2	402.08368	99.3	[M-H]-	58608	Glucosinolates
3109	7.423	Emodin	C15H10O5	271.01251	99.2	[M+H]+	148	Hydroxyanthraquinones

8813	15.751	18b-glycyrrhetinic	C30H46O4	471.33893	99.2	[M+H]+	58	
10559	12.908	Flavone base + 4O, O-MalonylHex	C24H22O14	535.10193	99.2	[M+H]+	19592	Flavone O-glycosides
11432	11.898	Epi-actephilol A	C35H32O8	581.21173	99.2	[M+H]+	27281	Phenanthrols
11819	5.146	thielavin B	C31H34O10	605.17072	99.2	[M+H]+	77327	Depsides and depsidones
5176	15.952	Villosinol	C23H22O8	425.22018	99.2	[M-H]-	244	Rotenones
6346	16.026	Gyrophoric acid	C24H20O10	467.09131	99.2	[M-H]-	942	
9672	11.515	quercetin-3-O-pentosyl-7-O-hexoside	C26H28O16	595.11627	99.2	[M-H]-	1253	Flavonoid-7-O-glycosides
2313	13.568	Acetosyringone	C10H12O4	235.08595	99.1	[M+H]+	10276	Alkyl-phenylketones
8442	14.277	3-Deoxycaryoptinol	C24H34O7	457.233	99.1	[M+H]+	29015	Furofurans
11931	9.752	apigenin 6,8-digalactoside	C27H30O15	612.18384	99.1	[M+H]+	96092	Flavonoid 8-C-glycosides
14523	18.704	Hederagenin base + O-dHex-Hex-Hex	C48H78O18	943.51221	99.1	[M+H]+	38	Triterpene saponins
1397	7.446	Diphenyl phosphate	C12H11O4P	249.03897	99.1	[M-H]-	232	Aryl phosphodiesters
1531	9.215	Phosphotyrosine	C9H12NO6P	260.03976	99.1	[M-H]-	42923	Phenylalanine and derivatives
2941	18.075	coronardine	C21H26N2O2	337.18713	99.1	[M-H]-	6270	Ibogan-type alkaloids
805	5.433	Aminocaproic acid	C6H13NO2	154.02795	99	[M+H]+	254	Medium-chain fatty acids
2240	7.379	Isoleucylvaline	C11H22N2O3	231.16371	99	[M+H]+	64750	Dipeptides
3457	18.368	Tris(2-chloroethyl)phosphate	C6H12Cl3O4P	284.95392	99	[M+H]+	14922	Trialkyl phosphates
10571	14.264	arctiin	C27H34O11	535.2099	99	[M+H]+	1053	Lignan glycosides
1635	15.176	HOMOCYSTINE	C8H16N2O4S2	267.04004	99	[M-H]-	18783	Alpha amino acids
988	6.485	Phthalamic acid	C8H7NO3	166.04227	98.9	[M+H]+	5845	Benzoic acids
2883	16.533	Cearoin	C14H12O4	262.09991	98.9	[M+H]+	54408	Benzophenones
8752	17.91	Euphol Acetate	C32H52O2	469.41129	98.9	[M+H]+	11	Triterpenoids

10282	8.5	Perseitol Heptaacetate	C21H30O14	524.2052	98.9	[M+H]+	42892	Monosaccharides
11211	13.124	Diatoxanthin	C40H54O2	566.42004	98.9	[M+H]+	542329	Triterpenoids
2191	16.633	Kaurenic acid	C20H30O2	301.2037	98.9	[M-H]-	19341	Kaurane diterpenoids
1392	6.876	D-(-)-3-PHOSPHOGLYCERIC ACID	C3H7O7P	186.99229	98.8	[M+H]+	345	Sugar acids and derivatives
8382	15.295	Phosphatidylcholine lyso alkyl 18	C26H56NO6P	540.39496	98.8	[M-H]-	8486	Monoalkylglycerophosphocholines
10996	15.665	Biflavonoid-flavone base + 3O and flavanone base + 2O + 1MeO	C31H22O10	555.13776	98.7	[M+H]+	48264	Biflavonoids
234	2.682	Threonic acid	C4H8O5	135.02171	98.7	[M-H]-	37269	Sugar acids and derivatives
2454	9.89	Pinoquercetin	C16H12O7	315.10788	98.7	[M-H]-	224231	Flavonols
4479	19.689	Constictic acid	C19H14O10	401.04007	98.7	[M-H]-	15150	
6717	27.351	beta-Sitosterol	C29H50O	397.3743	98.5	[M+H]+	82677	Stigmastanes and derivatives
1831	10.157	Feruloyl Lactate	C13H14O7	281.05615	98.5	[M-H]-	33593	Ferulic acid and derivatives
2312	7.403	FA 18:4+2O	C18H28O4	307.18103	98.5	[M-H]-	13907	Oxidized fatty acids
6249	17.635	4-hydroxyglucobrassicin	C26H44O9	463.06201	98.5	[M-H]-	8588	Medium-chain fatty acids
13808	17.55	MalonylHexsose + MalonylHexsose-deoxyHexose + C20H32	C44H68O22	947.39471	98.5	[M-H]-	57	Nicotianosides
5687	3.869	rosmarinic acid	C18H16O8	361.10187	98.4	[M+H]+	5158	Coumaric acids and derivatives
6086	7.202	matairesinol	C20H22O6	376.0564	98.4	[M+H]+	22852	Dibenzylbutyrolactone lignans
8044	7.784	neosolaniol	C21H28O9	442.19806	98.4	[M+H]+	1523	Trichothecenes

13134	12.746	Flavonol base + 3O, O-Hex, O-MalonylHex	C30H32O19	697.14868	98.3	[M+H]+	19025	Flavonol O-glycosides
13767	17.617	hyodeoxycholic acid	C24H40O4	785.57764	98.3	[M+H]+	12	
14371	4.993	camelliquercetiside C	C41H44O22	889.26349	98.3	[M+H]+	7519	Flavonoid-3-O-glycosides
566	13.121	Isoxanthopterin	C6H5N5O2	178.02757	98.2	[M-H]-	85284	Pterins and derivatives
2226	18.669	CYTIDINE 2',3'-CYCLIC MONO-PHOS-PHATE	C9H12N3O7P	304.04352	98.2	[M-H]-	153220	2',3'-cyclic pyrimidine nucleotides
6612	19.575	Neoglucobrassicin (1-Methoxy-3-indolylmethyl glucosinolate)	C26H44O9	477.03812	98.2	[M-H]-	49170	Medium-chain fatty acids
10600	15.994	Laccaic Acid A	C26H19NO12	538.08685	98.1	[M+H]+	266	
11988	11.419	Debromoaplysiatoxin	C32H48O10	615.25214	98.1	[M+H]+	39832	Macrolides and analogues
14151	14.529	Scytophycin E	C45H75NO12	844.54236	98.1	[M+H]+	18	Diterpene lactones
14177	18.179	Nodularin	C41H60N8O10	847.28339	98.1	[M+H]+	11	
3525	3.429	XANTHOSINE 5'-MONOPHOSPHATE	C10H13N4O9P	363.02423	98.1	[M-H]-	28331	Purine ribonucleoside monophosphates
3674	14.726	Didymic acid	C22H26O5	369.15714	98.1	[M-H]-	152631	
9058	9.241	quercetin-3-O-pentosyl(1-2)pentoside	C25H26O15	567.09595	98.1	[M-H]-	499	Flavonoid-3-O-glycosides
5691	18.942	Nonyl glucosinolate	C16H31NO9S2	444.1308	98	[M-H]-	8979	Glucosinolates
7249	11.303	Confluentic acid	C28H36O8	499.21683	98	[M-H]-	790	
3692	10.006	Norstictic Acid	C18H12O9	370.99805	97.9	[M-H]-	18141	Diarylethers
5252	5.945	methyl dichloroasterrate	C18H16Cl2O8	429.00476	97.9	[M-H]-	38183	Diphenylethers
3652	17.848	HYOSCYAMINE	C17H23NO3	290.26208	97.8	[M+H]+	16819	Tropane alkaloids
1372	5.22	Methylxanthoxyli	C11H14O4	248.95775	97.8	[M-H]-	13914	Alkyl-phenylketones
11176	11.706	S4:17(P3:15/F1:2)	C29H48O15	681.28247	97.7	[M-H]-	30140	Saccharolipids

2118	4.538	Nonic Acid	C9H16O4	227.01166	97.6	[M+H]+	92111	Medium-chain fatty acids
1149	2.322	2-QUINOLINECARBOXYLIC ACID	C10H7NO2	174.07118	97.5	[M+H]+	100205	Quinoline carboxylic acids
14461	2.76	Ursolic Acid	C30H48O3	913.28265	97.5	[M+H]+	20017	Triterpenoids
511	7.562	N-Methylthreonine	C5H11NO3	134.09258	97.4	[M+H]+	897	L-alpha-amino acids
13805	7.581	forsythoside B	C34H44O19	795.19269	97.4	[M+H]+	18665	Oligosaccharides
1099	18.8	Porphobilinogen	C10H14N2O4	225.07669	97.4	[M-H]-	7694	Aralkylamines
7036	16.026	Glucohirsutin	C16H31NO10S3	492.11511	97.4	[M-H]-	3301	Alkylglucosinolates
2518	11.236	Flavanone base + 6O	C15H12O8	319.05759	97.3	[M-H]-	16059	Flavanone O-glycosides
2579	16.518	Cytidine-5'-monophosphate	C9H14N3O8P	322.05627	97.3	[M-H]-	33659	Pyrimidine ribonucleoside monophosphates
12896	4.274	Biflavonoid-flavone base + 3MeO and flavone base + 3MeO	C40H34O10	675.24274	97.2	[M+H]+	625	Biflavonoids
3379	6.833	Virensic acid	C18H14O8	357.04584	97.1	[M-H]-	12869	
8235	5.803	Acetylloboridin	C25H28O13	535.12488	97	[M-H]-	1503	Phenolic glycosides
350	11.694	Threitol	C4H10O4	123.07771	96.9	[M+H]+	271902	Sugar alcohols
6208	21.697	monoolein	C21H40O4	379.32526	96.9	[M+H]+	20557	1-monoacylglycerols
10235	10.882	myricetin-3-O-hexosyl(1-2)deoxyhexoside	C27H30O17	625.09729	96.9	[M-H]-	5646	Flavonoid-3-O-glycosides
6003	6.86	Norrhizocarpic acid	C27H21NO6	454.10428	96.8	[M-H]-	461	
12819	3.914	Enterobactin	C30H27N3O15	670.13434	96.6	[M+H]+	468	
376	6.691	(S)-DIHYDROOROTATE	C5H6N2O4	157.01184	96.6	[M-H]-	6404	Alpha amino acids and derivatives
6389	18.898	asterric acid	C17H16O8	387.03427	96.5	[M+H]+	8727	Diphenylethers
10581	11.63	Gossypol	C30H30O8	536.24249	96.4	[M+H]+	718	Sesquiterpenoids
973	7.197	Propanil	C9H9Cl2NO	216.01247	96.4	[M-H]-	7154	Anilides

157	2.603	Gyromitrin	C4H8N2O	101.05724	96.3	[M+H]+	6149	N-alkylated hydrazones
10869	17.67	beta-Nicotinamide adenine dinucleotide	C21H27N7O14P	662.12006	96.3	[M-H]-	17252	(5'->5')-dinucleotides
12455	17.768	Aconitine	C34H47NO11	646.30334	95.8	[M+H]+	8642	Alkaloids
10196	11.987	LPC 18:2	C26H50NO7P	520.32471	95.7	[M+H]+	793	Lipids
2837	13.086	Isopongaflavone	C21H18O4	333.09833	95.7	[M-H]-	7386	Pyranoflavonoids
6021	2.013	Riboflavin-5'-monophosphate	C17H21N4O9P	455.08261	95.7	[M-H]-	117327	Flavin nucleotides
6770	13.148	CYTIDINE 5'-TRIPHOSPHATE	C9H16N3O14P3	481.99197	95.7	[M-H]-	16337	Pyrimidine ribonucleoside triphosphates
814	4.6	Cysteine S-	C3H7NO5S2	199.98454	95.5	[M-H]-	20997	S-sulfo-L-cysteines
1544	4.446	Sorbitol-6-phosphate	C6H15O9P	261.02298	95.5	[M-H]-	7300	Monosaccharide phosphates
13772	8.052	Flavone base + 4O, C-Hex-FeruloylHex	C37H38O19	787.18451	95.4	[M+H]+	102918	Ferulic acid and derivatives
933	4.269	2-Deoxyribose 5-phosphate	C5H11O7P	213.00162	95.4	[M-H]-	33977	Pentose phosphates
3651	11.694	paulownin	C20H18O7	369.01511	95.4	[M-H]-	92	Furanoid lignans
371	2.825	5-METHYLCYTOSINE HYDROCLORIDE	C5H7N3O	126.05062	95.3	[M+H]+	733292	Hydroxypyrimidines
2641	20.383	Bauerine B	C12H8Cl2N2	250.99641	95.3	[M+H]+	22007	Beta carbolines
1131	7.539	Dihydroresveratrol	C14H14O3	229.07158	95.3	[M-H]-	9535	Stilbene glycosides
12428	3.981	Flavin adenine dinucleotide	C27H33N9O15P	784.12543	95.3	[M-H]-	11099	Flavin nucleotides
10659	12.616	salviaflaside	C24H26O13	540.18835	95.1	[M+H]+	15733	Phenolic glycosides
8222	5.914	UDP-xylose	C14H22N2O16P	535.02014	95.1	[M-H]-	333	Pyrimidine ribonucleoside diphosphates

8905	7.335	Isoflavone base + 1O, 1MeO, O-Pen-Hex	C27H30O13	561.14294	95.1	[M-H]-	14449	Isoflavone O-glycosides
1367	4.743	Clothianidin	C6H8ClN5O2S	247.9854	95	[M-H]-	249564	2,5-disubstituted thiazoles
366	7.484	Protocatechuic acid	C7H6O4	153.02081	94.8	[M-H]-	102256	Hydroxybenzoic acid derivatives
6504	7.719	Succinprotocetraric acid	C22H18O12	473.04031	94.6	[M-H]-	6837	
11054	13.829	CEFPODOXIME PROXETIL	C21H27N5O9S2	558.11328	94.5	[M+H]+	14277	
3263	17.585	Vincamine	C21H26N2O3	353.02441	94.3	[M-H]-	301	Eburnan-type alkaloids
6830	3.735	Uridine 5_-triphosphate	C9H15N2O15P3	484.12283	94.3	[M-H]-	4633	Pyrimidine ribonucleoside triphosphates
13903	2.982	Xanthorhamnin	C34H42O20	809.14984	94.2	[M+H]+	10661	Flavonoid-3-O-glycosides
8644	6.305	Biflavonoid-flavone base + 3O and flavone base + 2O + 1MeO	C31H20O10	551.07776	94	[M-H]-	68844	Biflavonoids
9396	12.691	Tricoumaroyl spermidine	C34H37N3O6	582.23737	94	[M-H]-	391	Coumaric acid and derivatives
433	15.218	trans-4-Coumaric acid	C9H8O3	163.04074	93.9	[M-H]-	1684	Hydroxycinnamic acids
10049	29.728	Hexosyl LPE 16:0	C27H54NO12P	614.30615	93.7	[M-H]-	15730	Lipids
223	2.745	D-(+)-Malic acid	C4H6O5	133.0155	93.5	[M-H]-	514816	Beta hydroxy acids and derivatives
176	1.892	Choline	C5H14NO	104.1054	93.4	[M]+	8843927	Cholines
2012	1.821	Securinine	C13H15NO2	218.03844	93.4	[M+H]+	116740	Indolizidines
12715	7.127	Flavonol base + 4O, 1MeO, O-Hex, O-Hex, O-Hex	C34H42O23	817.17285	93.4	[M-H]-	22131	Flavonol O-glycosides

353	15.126	L-(-)-Mandelic acid	C8H8O3	151.04054	93.3	[M-H]-	390	Benzene and substituted derivatives
2611	19.992	Flavanone base + 2O, 1Prenyl	C20H20O4	323.1088	93.3	[M-H]-	2229	Prenylated chalcones
434	2.843	L-beta-Homoproline	C6H11NO2	130.08278	93.1	[M+H]+	10607	Pyrrolidines
13220	18.384	Furostane base -1H2O -2H + 1O, O-Hex, O-Pen-dHex	C44H70O17	869.42023	92.9	[M-H]-	64	Steroidal saponins
12929	11.586	Vitamin B12	3H88CoN14O1	678.26575	92.8	[M+H]+	521	Cobalamin derivatives
13157	18.384	MalonylHexsose + Hexsose-deoxyHexose + C20H32	C41H66O19	861.38062	92.8	[M-H]-	104	Nicotianosides
844	5.296	Orotic acid	C5H4N2O4	157.04414	92.5	[M+H]+	40477	Pyrimidinecarboxylic acids
10984	10.804	Purines I-IV gallate	C28H27NO11	554.14374	92.2	[M+H]+	28040	
11962	2.091	Eleutheroside E	C34H46O18	741.2312	92.2	[M-H]-	30156	Lignan glycosides
4789	20.383	Furosemide	:12H11ClN2O5:	330.99475	92.1	[M+H]+	49702	Aminobenzenesulfonamides
691	5.957	3-Indolepropionic acid	C11H11NO2	188.09203	92.1	[M-H]-	871	Indolyl carboxylic acids and derivatives
541	9.191	2-Isopropylmalic acid	C7H12O5	175.06285	92	[M-H]-	586483	Hydroxy fatty acids
11952	13.864	Phosphatidylethanolamine 16	C41H76NO8P	740.49341	92	[M-H]-	1333323	Phosphatidylethanolamines
223	1.656	HYPOTAURINE	C2H7NO2S	110.00644	91.9	[M+H]+	6079198	Sulfinic acids
13750	16.821	Soyasapogenol E base + O-HexA-Hex-dHex	C48H76O18	939.45532	91.9	[M-H]-	78	Triterpene saponins

10496	8.962	methoxy-myricetin-3-O-deoxyhexosyl-7-O-hexoside	C28H32O17	639.10535	91.7	[M-H]-	24562	Flavonoid-7-O-glycosides
5181	19.575	ADENOSINE 5'-DIPHOSPHATE	:10H15N5O10P	426.04318	91.5	[M-H]-	16218	Purine ribonucleoside diphosphates
12823	14.477	Phosphatidylcholine 17	C45H82NO8P	826.56189	91.5	[M-H]-	187667	Phosphatidylcholines
1416	24.586	Deoxyadenosine	C10H13N5O3	250.14766	91.4	[M-H]-	83438	Purine 2'-deoxyribonucleosides
12873	8.612	rescinnamine	C35H42N2O9	673.28094	91.3	[M+H]+	23751	Yohimbine alkaloids
5447	1.722	Hymenamide B	C42H54N8O10	352.87753	91.2	[M+H]+	19148	
1671	17.901	Apigenin	C15H10O5	269.04529	91.1	[M-H]-	15058	Flavones
6713	13.275	Glucomalcomiin	:17H23NO11S2	480.04236	91.1	[M-H]-	14180	Alkylglucosinolates
1460	1.967	Targinine	C7H16N4O2	189.13107	90.9	[M+H]+	807122	Arginine and derivatives
4936	12.881	Liquiritin	C21H22O9	417.11847	90.9	[M-H]-	426	Flavonoid O-glycosides
469	6.597	4-Hydroxyproline	C5H9NO3	132.04359	90.8	[M+H]+	236	Proline and derivatives
11878	13.012	Roseotoxin A	C31H53N5O7	608.37488	90.7	[M+H]+	172	Cyclic depsipeptides
749	2.013	D-Gluconic acid	C6H12O7	195.05228	90.7	[M-H]-	7704583	Medium-chain hydroxy acids and derivatives
4124	3.805	Dechlorodiploicin	C16H11Cl3O5	386.92563	90.5	[M-H]-	4904	
4742	7.353	Gangaleoidin	C18H14Cl2O7	411.0578	90.5	[M-H]-	187	Depsides and depsidones
13493	6.196	Eriodictyol-7-O-glucoside	C21H22O11	899.26544	90.5	[M-H]-	8074	Flavonoid-7-O-glycosides
9468	9.499	Malvidin-3-O-galactoside	C23H25O12	494.1189	90.4	[M+H]+	52182	Anthocyanidin O-glycosides
13811	10.288	Bipleiophylline	C47H46N4O8	795.37476	90.4	[M+H]+	18559	Pleiocarpaman alkaloids
1633	1.66	4-methylumbelliferone	C10H8O3	198.93661	90.3	[M+H]+	562729	7-hydroxycoumarins

712	2.218	D-(-)-quinic acid	C7H12O6	191.05682	90.2	[M-H]-	1552238	Quinic acids and derivatives
11608	13.362	Flavonol base + 4O, O-Hex, O-MalonylHex	C30H32O20	711.10767	90.1	[M-H]-	9621	Flavonol O-glycosides
12536	17.696	phosphatidylethanolamine (14:0/15:0)	C34H68NO8P	650.51031	89.7	[M+H]+	0	Phosphatidylethanolamines
532	3.074	ADENINE	C5H5N5	136.05855	89.6	[M+H]+	273441	
5305	3.588	Adenosine Monophosphate	C10H14N5O7P	348.06479	89.6	[M+H]+	874074	
14286	11.668	THEAFLAVIN DIGALLATE	C43H32O20	869.19684	89.6	[M+H]+	11197	Catechin gallates
3001	19.575	Caffeic acid hexoside	C15H18O9	341.06369	89.6	[M-H]-	51991	Caffeic acid and derivatives
4739	8.866	Leoidin	C18H14Cl2O7	411.04507	89.6	[M-H]-	137724	Depsides and depsidones
11072	7.74	Deacetoxy(7)-7-Oxokhivorinic Acid	C27H36O10	559.06372	89.5	[M+H]+	5803	Naphthopyrans
897	6.842	GALACTARATE	C6H10O8	209.00645	89.5	[M-H]-	21987	Glucuronic acid derivatives
1977	19.575	N-Fructosyl pyroglutamate	C11H17NO8	290.06357	89.4	[M-H]-	100577	N-Fructosyl amino acids
2682	5.863	cordycepin	C10H13N5O3	252.10423	89.3	[M+H]+	260993	
3130	5.377	Adenosine 3'-monophosphate	C10H14N5O7P	346.05643	89.3	[M-H]-	127350	Ribonucleoside 3'-phosphates
6730	6.834	Thymidine-5'-triphosphate sodium salt	:10H17N2O14P	480.95825	89.3	[M-H]-	1691	Pyrimidine 2'-deoxyribonucleoside triphosphates
1597	3.73	pencolide	C9H9NO4	264.98981	89.1	[M-H]-	15149	Alpha amino acids and derivatives
5622	3.583	GUANOSINE 5'-DIPHOSPHATE	:10H15N5O11P	441.99258	88.8	[M-H]-	10788	
482	3.735	Norleucine	C6H13NO2	132.13443	88.7	[M+H]+	460	Alpha amino acids
220	1.902	Aspartate	C4H7NO4	132.03165	88.7	[M-H]-	246080	Aspartic acid and derivatives
684	7.455	Gly-Leu	C8H16N2O3	187.10912	88.7	[M-H]-	11759	Peptides

2541	17.281	Dimethenamide ESA	C12H19NO5S2	320.03873	88.7	[M-H]-	12461	Heteroaromatic compounds
77	2.294	D-GLYCERIC ACID	C3H6O4	105.01974	88.5	[M-H]-	14581	Sugar acids and derivatives
748	2.063	Gluconate	C6H12O7	195.05215	88.5	[M-H]-	956993	Medium-chain hydroxy acids and derivatives
12538	2.725	Phosphatidylcholine alkenyl 16	C44H80NO7P	796.62561	88.5	[M-H]-	1637	1-(1Z-alkenyl),2-acyl-glycerophosphocholines
4847	4.876	Physodalic acid	C20H16O10	415.03217	88.4	[M-H]-	25556	
671	3.381	4-Guanidinobutyric acid	C5H11N3O2	146.08917	88.3	[M+H]+	122119	Gamma amino acids and derivatives
1001	8.113	Ephedrine	C10H15NO	166.1188	87.9	[M+H]+	869	Phenylpropanes
1745	8.052	Tryptophan	C11H12N2O2	205.09236	87.8	[M+H]+	4448190	Indolyl carboxylic acids and derivatives
1320	5.471	Tyrosine	C9H11NO3	182.07747	87.7	[M+H]+	1240118	Tyrosine and derivatives
902	8.766	Hydroxyferulic acid	C10H10O5	209.04724	87.7	[M-H]-	431	Hydroxyferulic acid and derivatives
431	4.858	L-5-Oxoproline	C5H7NO3	130.04756	87.5	[M+H]+	722639	Alpha amino acids and derivatives
825	6.202	Cyclo-prolylglycine	C7H10N2O2	155.0784	87.5	[M+H]+	2924	Alpha amino acids and derivatives
356	3.607	Isonicotinic acid	C6H5NO2	124.03564	87.3	[M+H]+	76027	Pyridinecarboxylic acids
5702	12.603	Glycitin	C22H22O10	445.02359	87.3	[M-H]-	24477	Isoflavonoid O-glycosides
3865	18.591	Ursinoic Acid	C15H16O5	298.99277	87	[M+H]+	9961	2,2-dimethyl-1-benzopyrans
9177	6.384	Phosphatidylcholine lyso 20	C28H48NO7P	572.30554	87	[M-H]-	103	2-acyl-sn-glycero-3-phosphocholines
10349	14.718	Isoflavone base + 1O, 2MeO, O-Hex+C7H12NO	C30H35NO11	630.18628	87	[M-H]-	25676	Isoflavone O-glycosides

700	1.924	L-Glutamine	C5H10N2O3	147.07359	86.8	[M+H]+	803791	Alpha amino acids
14340	14.632	Bacillibactin	C39H42N6O18	883.21582	86.8	[M+H]+	9876	Cyclic depsipeptides
13913	20.144	rebaudioside A	C44H70O23	965.37189	86.7	[M-H]-	14	Steviol glycosides
2797	1.938	sn-Glycero-3-phosphocholine	C8H21NO6P	258.10498	86.5	[M]+	1146981	Glycerophosphocholines
11995	12.922	Heme B	34H30FeN4O4]	616.15485	86.5	[M+H]+	87199	Metalloporphyrins
586	23.792	Mannose	C6H12O6	179.10689	86.5	[M-H]-	1216	Hexoses
1493	28.179	Palmitic Acid	C16H32O2	255.23296	86.4	[M-H]-	50894	
1190	6.91	Arecoline hydrobromide	C8H14BrNO2	234.0406	86.3	[M-H]-	4932	Alkaloids and derivatives
280	1.948	L-Glutamic acid	C5H9NO4	146.0459	86.2	[M-H]-	131198	Glutamic acid and derivatives
1566	13.232	Ferulic acid	C10H10O4	195.06122	86.1	[M+H]+	1562890	Hydroxycinnamic acids
14125	17.788	Licoricesaponin G2	C42H62O17	839.39624	86.1	[M+H]+	42	Triterpene saponins
339	2.171	L-(+)-tartaric acid	C4H6O6	149.01045	86.1	[M-H]-	448150	Sugar acids and derivatives
13645	19.463	Hexsose-deoxyHexose + Hexsose-deoxyHexose + C20H32	C44H74O20	921.42633	86.1	[M-H]-	17	Nicotianosides
476	5.911	Leucine	C6H13NO2	132.09877	86	[M+H]+	78873	Leucine and derivatives
1629	2.018	Galactose	C6H12O6	198.09491	86	[M+NH4]+	242554	Hexoses
4387	14.255	isorhamnetin	C16H12O7	317.06122	86	[M+H]+	55333	Flavonols
2921	10.271	Coumaroyl quinic acid	C16H18O8	337.09384	86	[M-H]-	187856	Quinic acid and derivatives
4682	1.849	Vanillic acid + O-sulfonateHex	C14H18O12S	409.01672	86	[M-H]-	58490	Sulfate containing metabolites
5578	4.386	butyrolactone II	C19H16O7	356.94241	85.9	[M+H]+	16872	
1869	6.136	Xanthosine	C10H12N4O6	283.06812	85.9	[M-H]-	68769	Purine nucleosides
553	9.655	6,7-DIHYDROXYCOUMARIN	C9H6O4	177.01881	85.6	[M-H]-	397	6,7-dihydroxycoumarins
687	5.373	N-Acetylglutamic acid	C7H11NO5	188.0575	85.5	[M-H]-	760	Glutamic acid and derivatives

4171	8.229	Barbatolic acid	C18H14O10	389.022	85.5	[M-H]-	145	
12222	5.884	Mesaconitine	C33H45NO11	632.27063	85.1	[M+H]+	64762	Alkaloids
13693	13.29	Furostane base + 2O, O-Hex, O-Hex	C39H66O16	773.38019	85.1	[M+H]+	355	Steroidal saponins
4919	9.323	kaempferol-3-O-pentoside	C20H18O10	417.04929	84.8	[M-H]-	223	Flavonoid-3-O-glycosides
4575	19.287	neobavaisoflavone	C20H18O4	323.12228	84.7	[M+H]+	716	
6901	8.581	Spongiacidin A	C11H9Br2N5O2	403.89316	84.7	[M+H]+	74	
438	4.784	N-Methyl-L-proline	C6H11NO2	130.09137	84.6	[M+H]+	136098	Proline and derivatives
4388	13.691	3-methylquercetin	C16H12O7	317.06128	84.5	[M+H]+	155967	
832	5.584	L-Histidine	C6H9N3O2	156.07249	84.4	[M+H]+	2875	Histidine and derivatives
344	15.375	NICOTINAMIDE	C6H6N2O	123.04158	84.3	[M+H]+	304670	Nicotinamides
6069	5.821	Epigallocatechin-3-Monogallate	C22H18O11	457.0484	84.3	[M-H]-	16219	
989	6.19	Methionine sulfoxide	C5H11NO3S	166.04301	84	[M+H]+	35511	Alpha amino acids
4212	8.866	S-HEXYL-GLUTATHIONE	C16H29N3O6S	390.14047	84	[M-H]-	38538	Oligopeptides
8379	23.863	LPC 16:0	C24H50NO7P	540.32983	84	[M+FA-H]-	41519	Lipids
350	4.846	Oxypurinol	C5H4N4O2	151.02742	83.9	[M-H]-	108880	Xanthines
1670	14.304	7,3',4'-Trihydroxyflavone	C15H10O5	269.04398	83.8	[M-H]-	77	Flavones
13921	13.153	acetyl CoA	23H38N7O17P3	810.08466	83.7	[M+H]+	100	Acyl CoAs
6694	6.468	geniposidic acid	C16H22O10	397.10535	83.6	[M+Na]+	234076	Iridoid O-glycosides
14084	13.233	Cephalomannine	C45H53NO14	832.30408	83.6	[M+H]+	385	Taxanes and derivatives
432	12.098	P-Coumaric acid	C9H8O3	163.04073	83.6	[M-H]-	11344	Hydroxycinnamic acids
2448	7.032	Benzoic acid + 2O, O-Hex	C13H16O9	315.07278	83.6	[M-H]-	303404	Phenolic glycosides
209	6.436	Glutaric acid	C5H8O4	131.03508	83.5	[M-H]-	23631	Dicarboxylic acids and derivatives

5524	8.795	CHLOROGENIC ACID	C16H18O9	355.09586	83.4	[M+H]+	1334262	Quinic acids and derivatives
5783	13.939	kaempferol 7-O-glucoside	C21H20O11	447.09442	83.4	[M-H]-	289624	
10993	12.508	Tricaprin	C33H62O6	554.76642	83.3	[M+H]+	98	Triacylglycerols
435	3.124	L-Pipecolic acid	C6H11NO2	130.08344	83.2	[M+H]+	7264391	Alpha amino acids
690	16.235	Cumarin	C9H6O2	147.04045	83.2	[M+H]+	3247	Coumarins and derivatives
174	1.98	4-AMINOBUTANOATE	C4H9NO2	104.06925	83.1	[M+H]+	1033791	Gamma amino acids and derivatives
3165	12.671	Naringenin	C15H12O5	273.06976	83.1	[M+H]+	237	
990	2.021	Methioninesulfoxide	C5H11NO3S	166.04845	82.9	[M+H]+	515159	Alpha amino acids
50	3.349	Lactic acid	C3H6O3	89.02562	82.9	[M-H]-	443058	Alpha hydroxy acids and derivatives
830	16.895	Sebacic acid	C10H18O4	201.11487	82.9	[M-H]-	32119	Medium-chain fatty acids
14013	5.426	Lanatoside C	C49H76O20	983.31183	82.8	[M-H]-	48324	Cardenolide glycosides and derivatives
472	8.059	2-Methylindole	C9H9N	132.07826	82.7	[M+H]+	56432	Indoles
511	12	Acetylleucine	C8H15NO3	172.09845	82.7	[M-H]-	20185	Amino acids
1447	15.756	6,4'-Dihydroxyflavone	C15H10O4	253.05112	82.7	[M-H]-	22	Flavones
295	3.048	L-NORVALINE	C5H11NO2	118.08312	82.4	[M+H]+	110952	L-alpha-amino acids
11905	12.432	Rutin	C27H30O16	611.15094	82.1	[M+H]+	180661	
9883	14.686	quercetin-3-O-pentosyl(1-2)acetylpentoside	C27H28O16	607.0849	82.1	[M-H]-	23032	Flavonoid-3-O-glycosides
479	5.615	D-Alloisoleucine	C6H13NO2	132.09924	82	[M+H]+	2382399	Isoleucine and derivatives
1989	2.748	Xanthotoxin	C12H8O4	217.02487	81.8	[M+H]+	3478	8-methoxypsoralens
13151	12.169	Sennoside B	C42H38O20	861.13385	81.8	[M-H]-	7576	Anthraquinones
839	8.083	Aconitic acid	C6H6O6	156.9818	81.7	[M+H]+	1059	Organic acids
3532	16.632	Sakuranetin	C16H14O5	287.12003	81.7	[M+H]+	128964	

2394	20.938	FA 18:2+2O	C18H32O4	311.22327	81.7	[M-H]-	42123	Oxidized fatty acids
165	5.347	1-Hexylamine	C6H15N	102.1012	81.6	[M+H]+	74911	Monoalkylamines
3615	11.116	3-O-Feruloylquinic acid	C17H20O9	367.10315	81.6	[M-H]-	487723	Quinic acids and derivatives
675	2.658	Acetylcholine	C7H16NO2	146.11353	81.5	[M]+	137549	Acyl cholines
2182	13.737	Hesperetin	C16H14O6	301.07529	81.4	[M-H]-	373	4'-O-methylated flavonoids
6750	9.849	RIBOFLAVIN	C17H20N4O6	399.12054	81.3	[M+Na]+	92403	Flavins
1712	23.338	Vasicinone	C11H10N2O2	203.10208	80.9	[M+H]+	40977	Quinazolines
6735	20.19	Ovoic acid	C25H22O10	481.06833	80.8	[M-H]-	24776	
14004	11.579	Berchemioside B	C50H62O20	981.31494	80.8	[M-H]-	8861	Oligosaccharides
9481	1.982	Podophyllin Acetate	C24H24O9	495.0112	80.7	[M+H]+	4986	Podophyllotoxins
1751	4.567	Azathioprine	C9H7N7O2S	275.99814	80.7	[M-H]-	78946	Diarylthioethers
2626	6.581	Matrine	C15H24N2O	249.19052	80.6	[M+H]+	2323	Matrine alkaloids
226	3.941	3-Pyridinemthanol	C6H7NO	110.05804	80.5	[M+H]+	570349	Pyridines and derivatives
727	17.475	trans-Cinnamic acid	C9H8O2	149.0574	80.5	[M+H]+	37318	Cinnamic acids
5491	18.051	Anileridine	C22H28N2O2	353.22391	80.5	[M+H]+	1350564	Phenylpiperidines
361	6.787	N,N-Dimethylsulfamide	C2H8N2O2S	125.01997	80.3	[M+H]+	37482	Sulfuric acid diamides
910	26.053	6-Methylcoumarin	C10H8O2	161.05525	80.3	[M+H]+	26264	Coumarins and derivatives
5797	2.407	isomaltulose	C12H22O11	365.10077	80.2	[M+Na]+	9103952	O-glycosyl compounds
1382	3.394	O-Phosphoserine	C3H8NO6P	185.98291	80.1	[M+H]+	1972	L-alpha-amino acids
14007	18.172	Glycyrrhizic Acid, Ammonium Salt	C42H62O16	823.40594	80	[M+H]+	155	
3281	7.71	3-Caffeoylquinic acid	C16H18O9	353.08945	80	[M-H]-	934323	Quinic acids and derivatives
